# Credible practice of modeling and simulation in healthcare: ten rules from a multidisciplinary perspective

**DOI:** 10.1186/s12967-020-02540-4

**Published:** 2020-09-29

**Authors:** Ahmet Erdemir, Lealem Mulugeta, Joy P. Ku, Andrew Drach, Marc Horner, Tina M. Morrison, Grace C. Y. Peng, Rajanikanth Vadigepalli, William W. Lytton, Jerry G. Myers

**Affiliations:** 1 Department of Biomedical Engineering and Computational Biomodeling (CoBi) Core, Lerner Research Institute, Cleveland Clinic, 9500 Euclid Avenue (ND20), Cleveland, OH 44195 USA; 2 InSilico Labs LLC, 2617 Bissonnet St. Suite 435, Houston, TX 77005 USA; 3 Department of Bioengineering, Clark Center, Stanford University, 318 Campus Drive, Stanford, CA 94305-5448 USA; 4 Oden Institute for Computational Engineering and Sciences, University of Texas at Austin, 201 E. 24th st, Austin, TX 78712 USA; 5 ANSYS, Inc, 1007 Church Street, Suite 250, Evanston, IL 60201 USA; 6 Division of Applied Mechanics, United States Food and Drug Administration, 10903 New Hampshire Avenue, Silver Spring, MD 20993 USA; 7 National Institute of Biomedical Imaging & Bioengineering, Suite 200, MSC 6707 Democracy Blvd5469, Bethesda, MD 20892 USA; 8 Department of Pathology, Anatomy and Cell Biology, Daniel Baugh Institute for Functional Genomics/Computational Biology, Thomas Jefferson University, 1020 Locust St, Philadelphia, PA 19107 USA; 9 State University of New York, Kings County Hospital, 450 Clarkson Ave., MSC 31, Brooklyn, NY 11203 USA; 10 Human Research Program, Cross-Cutting Computational Modeling Project, National Aeronautics and Space Administration - John H. Glenn Research Center, 21000 Brookpark Road, Cleveland, OH 44135 USA; 11 Committee on Credible Practice of Modeling, & Simulation in Healthcare, Interagency Modeling and Analysis Group and Multiscale Modeling Consortium, Bethesda, MD USA

**Keywords:** Credibility, Simulation, Healthcare, Verification, Validation, Computational modeling, Computer modeling, Reliability, Reproducibility

## Abstract

The complexities of modern biomedicine are rapidly increasing. Thus, modeling and simulation have become increasingly important as a strategy to understand and predict the trajectory of pathophysiology, disease genesis, and disease spread in support of clinical and policy decisions. In such cases, inappropriate or ill-placed trust in the model and simulation outcomes may result in negative outcomes, and hence illustrate the need to formalize the execution and communication of modeling and simulation practices. Although verification and validation have been generally accepted as significant components of a model’s credibility, they cannot be assumed to equate to a holistic credible practice, which includes activities that can impact comprehension and in-depth examination inherent in the development and reuse of the models. For the past several years, the Committee on Credible Practice of Modeling and Simulation in Healthcare, an interdisciplinary group seeded from a U.S. interagency initiative, has worked to codify best practices. Here, we provide Ten Rules for credible practice of modeling and simulation in healthcare developed from a comparative analysis by the Committee’s multidisciplinary membership, followed by a large stakeholder community survey. These rules establish a unified conceptual framework for modeling and simulation design, implementation, evaluation, dissemination and usage across the modeling and simulation life-cycle. While biomedical science and clinical care domains have somewhat different requirements and expectations for credible practice, our study converged on rules that would be useful across a broad swath of model types. In brief, the rules are: (1) Define context clearly. (2) Use contextually appropriate data. (3) Evaluate within context. (4) List limitations explicitly. (5) Use version control. (6) Document appropriately. (7) Disseminate broadly. (8) Get independent reviews. (9) Test competing implementations. (10) Conform to standards. Although some of these are common sense guidelines, we have found that many are often missed or misconstrued, even by seasoned practitioners. Computational models are already widely used in basic science to generate new biomedical knowledge. As they penetrate clinical care and healthcare policy, contributing to personalized and precision medicine, clinical safety will require established guidelines for the credible practice of modeling and simulation in healthcare.

## Background

Computational modeling and simulation has become increasingly popular in biomedical research and has found proven utility in healthcare. However, the ecosystem of biomedical simulation is fragmented and workflows among different biomedical disciplines exhibit large operational variations. These circumstances introduce difficulties in establishing and conveying the *credibility* of computational modeling and simulation outcomes. A common operational framework to provide a practical basis for the assessment of computational modeling studies used for scientific and clinical decisions is therefore an imperative need.

Computational modeling has been reliably used in traditional engineering disciplines to support product development and evaluation. The biomedical field, however, has been slower to adopt these approaches. In historically strong engineering disciplines, one can apply mathematical modeling through direct implementation of first principles and community-accepted frameworks to human-made systems with known system parameters. In biomedical fields, we are often tasked with reverse engineering comprehensive, and sometimes complex, systems made up of disparate elements with only a partial understanding of their properties or even their functions. The lack of fundamental first-principle approaches contributes to the challenge that medical practitioners face in developing confidence in model-supported results. Establishing the credibility of biomedical simulations is particularly challenging, as biomedical simulations are typically and often purposely built to cover particular spatial and temporal scales where acquiring direct measures is difficult, thus impeding our ability to establish accuracy by direct comparison. Despite the challenging nature of the task, establishing model credibility in this domain is particularly important given its potential for direct application to patient care. Leading government, health, academic, and private institutions around the world recognize that computational methods exhibit tremendous potential to support clinical research and decision-making in healthcare [1–5]. The subject of credibility has been of increasing interest to the modeling and simulation community for many years. In modeling and simulation literature, including those in healthcare, verification and validation have been considered the primary component of a model’s credibility. Thus, for the most part, equating the credibility of the practice to evaluation of validity of the model or model outputs. The present study takes a comprehensive approach by treating credibility as a term inclusive of validation but incorporating many other aspects that critically impacts the overall quality of the modeling and simulation practice.

There are a number of industry and government initiatives focused on establishing credibility in modeling [6, 7] as well as supporting the adoption of computational models as a trusted source of data. Organizations–including the European-based Avicenna Alliance [2], the industry-led Medical Device Innovation Consortium [3], the U.S. Food and Drug Administration (FDA) [4], and the United States Congress [5]–have specifically advocated for the use of in silico clinical trials, or trials based purely on computational methods to advance the development and evaluation of new medical devices, drugs, and interventions. Cited benefits of the in silico clinical trials approach include streamlined “regulatory evaluation of a medicinal product or medical device/medical intervention” [2], development of comprehensive “virtual patients” representing a continuum of information [3], accelerated innovations in clinical evaluations [4], and advancement of new devices and drug therapy applications [5]. With these developments, establishing frameworks for regulation of computational simulation has become a pressing need worldwide, e.g. as noted by the European Economic and Social Committee [8].

These suggestions focused on more mature or forward-looking application scenarios and did not propose guidelines for incorporating credible modeling and simulation practices into fundamental and translational research initiatives, such as those funded through the Interagency Modeling and Analysis Group since 2004 [9]. The community of funded researchers from these initiatives formed the Multiscale Modeling Consortium to promote the advancement of methodologies to address mechanistic multiscale modeling in biomedical, biological and behavioral systems. However, both the Interagency Modeling and Analysis Group and Multiscale Modeling Consortium encountered substantial skepticism from the clinical community on the trustworthiness of such models to inform healthcare practice. This initiated a multi-year discussion among the community to establish methods and guidelines for assessing model robustness, with an emphasis on verification, validation and uncertainty quantification [10–12]. Meanwhile, the National Research Council and the United States President’s Council of Advisors on Science and Technology outlined that healthcare benefits of advanced approaches, including computational modeling, often accrue once they are deemed sufficiently trustworthy both by researchers and caregivers [13, 14]. Drawing insights from NASA’s history on applying computational models for novel biomedical applications, the Interagency Modeling and Analysis Group/Multiscale Modeling Consortium community recognized that developing confidence in models and simulations required a holistic process that occurs over time and involves multiple intertwined activities.

As an example, consider the evaluation of a model to generate trust in using the model outputs for decision-making purposes. A common theme in the model and simulation credibility literature is that trust in a model depends strongly on the level of testability of the model and simulation [15–19]. Generally, this involves the validation, comparison and evaluation of differences in performance to an adequate referent, although verification [18], uncertainty quantification and sensitivity analysis [20] should also play a substantial part in the testing paradigm. Clearly testing a model and simulation performance with respect to a quantitative referent that is representative of the real-world system would provide compelling evidence to the credibility of the model and simulation [16, 17]. The engineering modeling and simulation literature is a useful resource for methods to perform validation comparisons and guidance in evaluating validation results [15, 21], including methods for evaluating with only subject matter expert opinion and for combining comparable validation factors of differing strengths. In combination with the model and simulation output, the extent to which the comparator referent represents the real-world system and the extent it covers the context of use of the model directly relates to the strength of the validation activity to influence model and simulation credibility.

Direct validation is often impossible for healthcare applications because it requires a comparator that matches the fidelity of the real-world system and its environment with respect to the intended application of the modeling and simulation products and their associated influence and consequence on resulting decisions and actions, i.e., the modeling and simulation context of use [22, 23]. The inability to perform direct validation hinders the broader acceptance and reliability of computational modeling in healthcare. However, indirect validation can be accomplished utilizing an additional comparator, for example an animal disease model. Of course, there may be significant differences between this comparator and the clinical situation, for example, in the environment experienced by animal models as compared to patients. These differences must be identified and evaluated in order to establish trust and confidence in the computer model within the stated context of use. This leads to a secondary challenge of reporting modeling and simulation processes and testing results with sufficient breadth and detail to communicate the applicability of the modeling and simulation in a healthcare setting. Further, even if direct validation and adequate reporting are performed, adoption of a computational model for decision-making would also need to consider the rigor and reproducibility of the underlying modeling and simulation activities. Computational models, with multiple levels of complexity and the potential to inform clinical and policy decisions, pose implementation challenges to the user community. The risks associated with inappropriate or ill-placed trust in the model outcomes, especially in time-critical situations, must be weighed appropriately by review and evaluation of available evidence with respect to the intended use of the model and the questions being asked of the model and simulation. This illustrates the need to formalize the communication of modeling and simulation practices consistent with the execution of such practices.

The body of evidence the developer provides directly influences the ability to communicate aspects of credibility [16, 24]. Evidence of credibility in healthcare-related modeling and simulation should bridge between developers and decision makers by communicating aspects of credibility in a manner that is independent of the decision maker’s involvement with the underlying development. In an ideal development situation, the decision maker, by being an integral part of the development process, establishes inherent “buy-in” to the design, assumptions, testing and evaluation of the model performance. However, decision making and user involvement is not guaranteed in the healthcare domain, where models can be developed and used for a large variety of applications (clinical decision making, hypothesis generation for experiment design, policy, communication) with a specific stakeholder in mind, i.e., clinicians, but not always with that user community’s involvement. In that sense, there should be a common practice to capture and communicate this critical ancillary evidence the healthcare community might expect from research driven model development activities. This further infers the need for a general, and customizable, framework that is inclusive of all activities of modeling and simulation in establishing modeling simulation credibility not only for scientific research but also for translation to a clinical environment.

## Roadmap to establish guidance on modeling and simulation practices

**Fig. 1:**
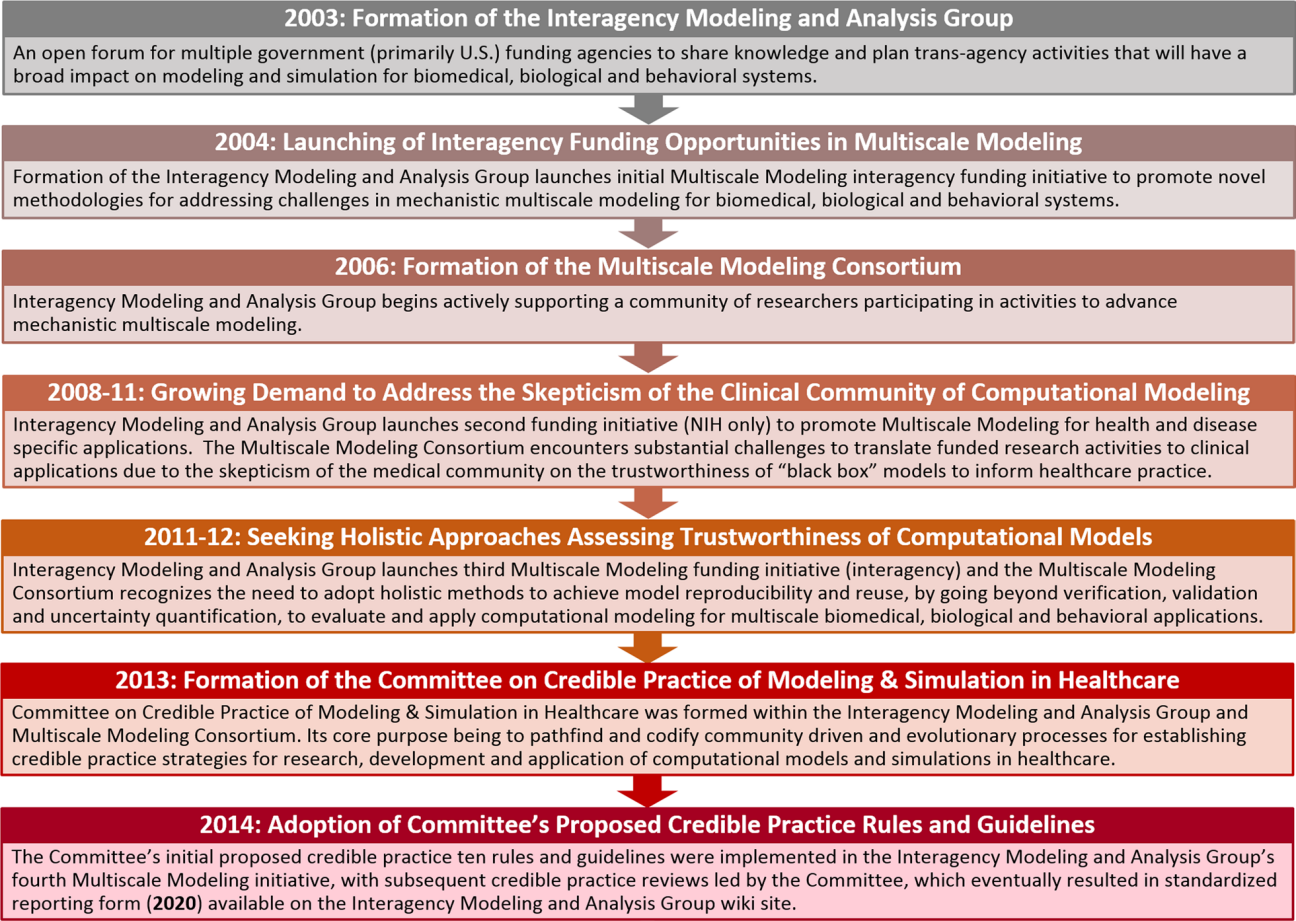
The research community events leading to the formation of the Committee on Credible Practice of Modeling and Simulation in Healthcare. The mission of the Interagency Modeling and Analysis Group and the Multiscale Modeling Consortium [9] is to share novel methodologies to cross spatial and temporal scales in biomedical, biological and behavioral systems, by promoting model reproducibility and reuse [26]. To achieve this goal, the end user must be first convinced to use each model through evaluating transparent credible practice rules for modeling and simulation, carried out by each modeler

To provide guidance into this complex process, we formed the Committee on Credible Practice of Modeling and Simulation in Healthcare (hereafter referred to as the Committee) in 2013 (See Fig. 1). Our mission is to develop a holistic and broadly applicable approach to understand, establish, and describe guidance and standards that enable the credible and reliable use of biomedical modeling and simulation in the practice of healthcare and translational research. The Committee is a working group of the Interagency Modeling and Analysis Group and the Multiscale Modeling Consortium [25], which are organized by the U.S. National Institutes of Health (NIH) in collaboration with academic researchers and multiple U.S. government agencies to promote the advancement of computational methods in healthcare practice and translational research. In launching this initiative, the founding Committee members established the following definitions as a general consensus of what it means to engage in Credible Practice of Modeling and Simulation in Healthcare [25]:

- Credible: Dependable, with a desired certainty level to guide research or support decision-making within a prescribed application domain and intended use; establishing reproducibility and accountability.
- Practice: Any activity involving the development, solution, interpretation and application of computational representations of biological, environmental and man-made systems and their interaction thereof.
- Modeling: Virtual, in silico, representation of system(s) of interest in a usable form in order to provide descriptive and predictive metrics for timely and systematic exploration of said system(s).
- Simulation: Computational solution of models that quantify descriptive and predictive metrics of system(s) of interest, including related post-processing efforts to calculate these metrics from raw analysis results.
- Healthcare: Any activity involving development, maintenance, advancement, or administration of medical care, including research, diagnosis, risk assessment, prevention, therapy, rehabilitation, surgery, intervention design, and regulation.

This paper presents the results from the Committee’s efforts to establish the “Ten Rules for Credible Practice of Modeling and Simulation in Healthcare.” The Ten Rules were established through continuous engagement and discussions within the Committee and with the broader biomedical research community since the Committee’s inception in 2013 [25]. Using a two phased approach, the Committee assessed factors related to the credibility of modeling and simulation activities within and between implementing disciplines.

In the first phase, the Committee Co-Chairs assembled three task teams from the Committee roster and asked them each to identify the top ten rules of credible practice from an initial set of 26 rules (Table 1). The task teams’ organization followed the Committee estimated proximity to clinical applications of modeling and simulation in healthcare: (1) those weighing towards mathematics and computation, (2) those who have a vested interest in the end-use of modeling and simulation, and (3) those with an inclination towards standards, guidance, evaluation and regulation (Additional file 2: Table SM-1). Each team also consisted of multidisciplinary members with respect to scientific and/or clinical background to minimize bias towards any particular discipline. This mix of subject matter expertise provided a comprehensive and balanced input regarding the goals and objectives of the rules to be developed. The three teams came up with different prioritizations of the rules, highlighting how discipline, application purpose, and background can noticeably influence perspectives.

**Table 1:** The initial 26 proposed rules of good practice surveyed within the Committee

Use version control	Use credible solvers	Explicitly list your limitations
Report appropriately	Document your code	Provide examples of use
Practice what you preach	Develop with the end user in mind	Attempt validation within context
Follow discipline-specific guidelines	Attempt verification within context	Attempt uncertainty (error) estimation
Make sure your results are reproducible	Define your evaluation metrics in advance	Conform to discipline-specific standards
Be a discipline-independent/ specific example	Learn from discipline-independent examples	Use appropriate data (input, validation, verification)
Define the context the model is intended to be used for	Perform appropriate level of sensitivity analysis within context of use	Use consistent terminology or define your terminology
Get it reviewed by independent users/developers/members	Provide user instructions whenever possible and applicable	Use traceable data that can be traced back to the origin
Disseminate whenever possible (source code, test suite, data, etc.)	Use competition of multiple implementations to check and balance each other	

The Committee Co-Chairs curated the ranking, elaboration, and consolidation of these rules to establish an initial Committee recommendation of the Ten Rules of credible practice of modeling and simulation in healthcare [27]. Rules with similar outcomes in terms of their role in the assessment of credibility, or those that may enhance each other, were grouped into a single consolidated rule. Unique perspectives from the teams and individuals were also noted because they might reflect a discipline-specific or context-specific need to establish credible practice of modeling and simulation.

In the second phase, a public survey—with worldwide participation and a wide variety of perspectives and background in healthcare modeling and simulation—was conducted from August 15, 2014 to April 15, 2015. Participants provided a relative ranking of the common modeling and simulation practices, an updated list of 32 potential rules synthesized during the discussions of the three task teams (Additional file 2: Table SM-2), to guide the final selection of the ten rules [28, 29]. Additional details of the Committee approach and analysis to reach the final ten rules can be found elsewhere [25, 27, 28].

## Recommendations for credible modeling and simulation practices in healthcare

Here we present the synthesis of the Committee’s efforts to develop the Ten Rules for credible practice of modeling and simulation in healthcare from the comparative analysis of the Committee’s modeling and simulation discipline perspective and the stakeholder community survey. The Ten Rules for credible practice of modeling and simulation presented evolved from ten rules identified by initial discussions within the Committee and four overlapping credibility concepts that were determined through an initial evaluation of the public survey [28]. Since then, the Committee perspective and the community perceptions were consolidated further. The Ten Rules have been hardened by their incorporation into funding mechanisms [30–34] and continuous discussions with the investigators and funding agency representatives [9] who use these rules. They represent a robust and holistic approach that not only encompasses rigorous verification and validation practices but also adoption of activities intended to enhance the practice as a whole. They also support the communication of important, and potentially neglected, evidence of credibility inherent in the development process not presented in early work.

These Ten Rules seek to establish a unified conceptual framework to design, implement, evaluate, and communicate the activities, products, and outcomes of the modeling and simulation life-cycle in a fashion that is agnostic to the biomedical science and clinical care domain. The rules, as detailed in the text and summarized in Table 2, may appear to be common sense guidelines, but provide a unified framework for both new modelers and seasoned practitioners. In the healthcare ecosystem, diverse expertise levels in modeling and simulation drives the need to facilitate communication on implementation or to understanding simulations predictions between stakeholders, e.g., between the developer of the models, practitioners of the modeling and simulation practice, users of the models, and/or users and decision makers of the knowledge generated by the modeling and simulation practice, such as clinicians and policy makers. They are also good reminders of the breadth of considerations that needs to be accounted for during model development and deployment, as lacking consideration of one or more will likely handicap the credibility of a modeling and simulation activity. Computational models have widespread utility to generate new biomedical knowledge and are now penetrating clinical care and healthcare policy through individualized care and regulatory support, respectively. As a result, scientific rigor and clinical safety increasingly require established credible practices in modeling and simulation.

**Table 2:** The Committee’s Ten Rules of credible practice of modeling and simulation in healthcare

Rule		Description
1.	Define context clearly	Develop and document the subject, purpose, and intended use(s) of the model or simulation
2.	Use contextually appropriate data	Employ relevant and traceable information in the development or operation of a model or simulation
3.	Evaluate within context	Perform verification, validation, uncertainty quantification, and sensitivity analysis of the model or simulation with respect to the reality of interest and intended use(s) of the model or simulation
4.	List limitations explicitly	Provide restrictions, constraints, or qualifications for or on the use of the model or simulation for consideration by the users or customers of a model or simulation
5.	Use version control	Implement a system to trace the time history of modeling and simulation activities including delineation of each contributors’ efforts
6.	Document appropriately	Maintain up-to-date informative records of all modeling and simulation activities, including simulation code, model mark-up, scope and intended use of modeling and simulation activities, as well as users’ and developers’ guides
7.	Disseminate broadly	Share all components of modeling and simulation activities, including simulation software, models, simulation scenarios and results
8.	Get independent reviews	Have the modeling and simulation activity reviewed by nonpartisan third-party users and developers
9.	Test competing implementations	Use contrasting modeling and simulation implementation strategies to check the conclusions of different strategies against each other
10.	Conform to standards	Adopt and promote generally applicable and discipline specific operating procedures, guidelines, and regulations accepted as best practices

**Fig. 2:**
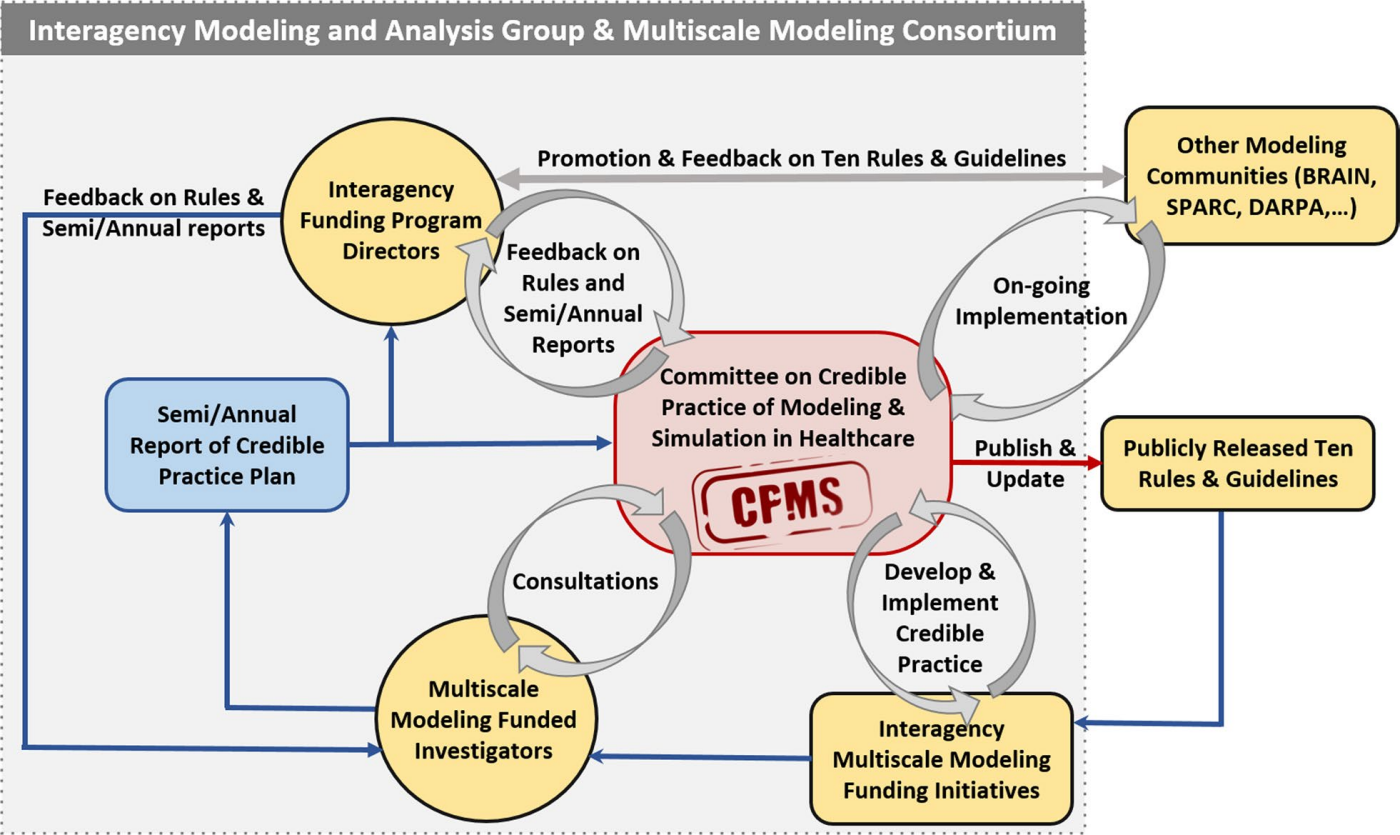
Process for maintaining and evolving the Ten Rules for credible practice in model and simulations in healthcare at the time of the development of this manuscript. The Committee utilizes an iterative process to ensure the Ten Rules and its supporting materials remain relevant and useful. Government agencies have incorporated the Ten Rules into their funding solicitations to guide applicants on how to develop a credible practice plan [30–34]. Informal mechanisms (gray arrows), such as discussions with the funded investigators and program directors of these solicitations, provide invaluable feedback to incorporate into the Committee’s guidelines. Within the Interagency Modeling and Analysis group, funded investigators also submit semi-annual reports, which include updates on how their projects fulfill the Ten Rules (now available as a online form that can be continuously updated on the Interagency Modeling and Analysis Group wiki site [9]). Through this formal process (blue arrows), the Committee receives additional feedback for improving the Ten Rules and guidelines

However, we are fully aware these Ten Rules of credible practice are not static, just as scientific and clinical methods are not static. With the growing use of the rules, we have received continuous feedback from the research community on ways to improve the communication and application of the rules. For example, recent developments have demonstrated the need to establish a rubric customized for the Multiscale Modeling Consortium to help modelers articulate the level of conformance necessary to be achieved for each rule [35]. This is because the degree to which each rule should be and can be applied will vary dramatically depending on the context of use, state of biomedical knowledge and modeling methodologies used. Therefore, we have adopted an iterative approach for continuously updating the Ten Rules and supporting guidelines (Fig. 2).

### Rule 1—define context clearly

Rule 1 impacts the implementation of six other rules, so we advocate establishing a clear definition of the modeling and simulation context of use in the earliest phases of the planning, development and implementation of the modeling and simulation efforts. A well-articulated context of use statement facilitates the ability of researchers and developers to use appropriate data (Rule 2), implementation techniques (Rule 9), and evaluation methods (Rules 3 and 8) to plan and develop the modeling and simulation activities. It can also help end-users gain quick and accurate insight into the utility, scope and limitations of the modeling and simulation (Rule 4).

A complete context of use, as graphically represented in Fig. 2, defines the following three elements:

- Domain of use: the domain(s) of healthcare that the specific version of the modeling and simulation (Rule 5) under consideration is intended to inform.
- Use capacity: capacity to which the modeling and simulation can be used, including metrics that are targeted for predictions and the potential consequences of the use.
- Strength of influence: the importance of the modeling and simulation to draw conclusions or decisions within the stated Domain of Use and Use Capacity (Rule 4).

As the context of use prescribes clearly defining the expected purpose and application of the model and simulations, it contributes to the implementation of all aspects of the model and simulation life-cycle. In practice, developers should seek to clearly delineate (1) the descriptions of the real-world system being modeled, (2) the level of agreement a model would need to provide to influence decisions, (3) the concepts behind the model and its use in simulations, and 4) key processes that must be captured for the model to be representative of the real-world system and its interactions with the modeled environment. An example of how one might practice Rule 1 is provided in the work of Pennline and Mulugeta [17] and their related activities, which are summarized in the Additional file 1: Example 1. For their modeling and simulation practice, they define the Domain of Use, Use Capacity and intended Strength of Influence of their bone physiology computational model. Further guidance on describing the context of use can be obtained from practical applications documents published in aerospace disciplines [36].

### Rule 2—use contextually appropriate data

**Fig. 3:**
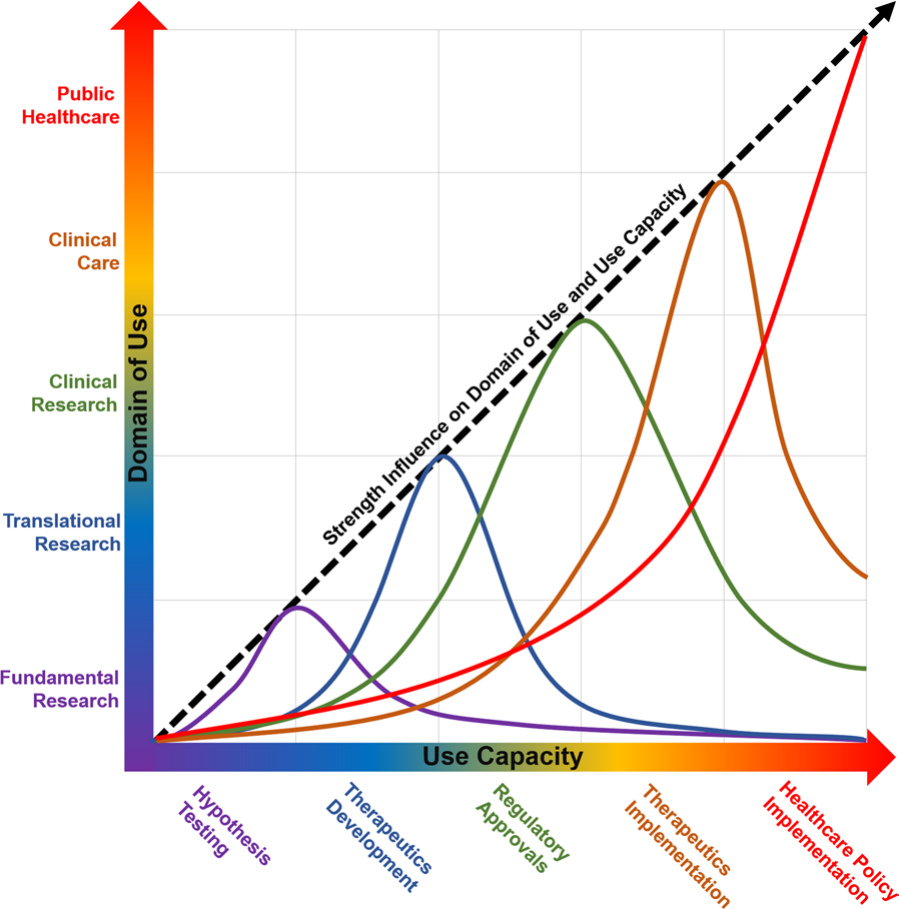
Relation between Model and Simulation Domain of Use, Use Capacity and Strength of Influence. Model and Simulation developed for a specific Domain of Use will typically have the greatest Strength of Influence within a commensurate range of Use Capacity. It may, however, be able to provide inference data for other Use Capacity areas. For example, an modeling and simulation framework specifically intended for translational research (blue line) in pharmaceuticals is likely to have the highest Strength of Influence in therapeutics development (e.g. new drug development). Similarly, a highly vetted epidemiological modeling and simulation to analyze the long-term effect(s) of an FDA-approved vaccine on public health (red line) is likely to be most credible for informing healthcare policy and preventative therapeutics implementation. The Strength of Influence of these examples would likely differ should the Use Capacity involve applications related to regulatory approval, therapeutics development, and hypothesis testing

Comprehensively conforming to Rule 2 means that (1) all the data used in the development, operation, and evaluation of the modeling and simulation are traceable to their original source, (2) the data’s relevance to the stated context of use is well articulated, and (3) ideally, the Domain of Use experts that are not modeling and simulation practitioners can understand which and how the data were used. This rule is closely linked to other rules to clearly define the model context (Rule 1), evaluate the modeling and simulation within context (Rule 3), and explicitly account for the modeling and simulation limitations (Rule 4). Development and operation of a modeling and simulation with a well-specified context of use (Rule 1) needs to employ data with established relevance to the context of use. Ideally, the scope, type, size, breadth, and other characteristics of the data should be aimed towards maximizing the Use Capacity (i.e., generalizability) and Strength of Influence on the Domain of Use (Fig. 3). To achieve these aims, the data may be taken from a wide range of sources, including different animal models, experimental testing and human cases. An increasingly important modeling and simulation data consideration is the recognition of sex as a significant biological variable that should be more frequently incorporated in study design, analysis, and data collection [37]. Furthermore, modeling and simulation efforts aimed at personalized and precision medicine necessitate the collection and use of individual data to formulate and parameterize patient-specific models.

In addition to the use of relevant data, Rule 2 calls for the employment of traceable information in the development and operation of modeling and simulation, which is in line with FAIR principles for scientific data management (**F**indable, **A**ccessible, **I**nteroperable, **R**eusable) [38]. Note that in some cases, while the data employed are traceable, there may be limited or no availability for independent third-party evaluation or for testing competing implementations (Rules 8 and 9, respectively), e.g., when using proprietary data in therapeutic development. In these cases, credible practice of modeling and simulation may be achieved via evaluation by the appropriate regulatory bodies governing the domain of use, for example, the FDA in the case of drug development. The availability and use of the appropriate data for modeling and simulation development has a direct bearing on the ability to test competing implementations (Rule 9). For example, different implementations of the modeling and simulation may place divergent requirements on the data, such as on cellular versus molecular resolution or the number of time points. An example of contextual relevance of data used for model development and its evaluation can be seen in the work by Rajagopal et al. [39] (also see Additional file 1: Example 2). With the goal of predicting musculoskeletal load sharing during normal gait of healthy young adult individuals, they rely on cadaver anatomical data augmented with data from young human subjects for model development. They track gait data of a young adult subject to predict muscular activation and evaluate against muscle electromyography patterns.

An important consideration for healthcare modeling and simulation activities related to Rule 2 is the quality of the referent data used to evaluate the model. For biological structures and in clinical domains, data can be noisy, highly variable, and/or incomplete, indicating challenges in terms of their acceptance as referent. Assessment of data’s suitability for this purpose, is an important consideration that builds upon Rule 2 and impacts any activity related to validation. Guidance available in other domains, such as [36], can be helpful in this regard.

### Rule 3—evaluate within context

The continuous evaluation of modeling and simulation within different contexts of healthcare is necessary for these tools to become widely accepted. This process is one we have observed in the automotive, aerospace, and nuclear industries, and emerges as an important step when informing high stakes decisions using modeling and simulation, e.g., those decisions related to directing patient care or public policy. Ideally, evaluation of any modeling and simulation should be ingrained into the iterative development and testing process, accompanied by evidence of efficacy in their respective contextual domains, which will drive the eventual acceptance of these technologies into the broader society [40–42]. Similarly, for society to fully realize the potential of modeling and simulation to positively impact healthcare, it is imperative to apply contextually robust evaluation methods.

The evaluation process focuses on the practices of verification, validation, uncertainty quantification, and sensitivity analysis:

- Verification is the process of determining that the computational modeling and simulation accurately represents the underlying mathematical model and its solution [43, 44]. A domain-specific example of code and calculation verification requirements would be one developed in accordance with ASME V&V10-2006 [44].
- Validation of the modeling and simulation is the process of determining the degree to which the model is an accurate representation of the real world from the perspective of its context of use [44]. Due to the abstraction needed to represent complex biological behaviors within modeling and simulation, some deviation from the real system performance should always be expected. Therefore, the required level of accuracy should be considered in view of the usefulness of the modeling and simulation as dictated by its context of use.
- Uncertainty quantification of the modeling and simulation is needed to characterize the pertinent variability in the model and comparator and to quantify their effect on the simulation outcomes [44]. These uncertainties emerge from data, modeling assumptions, numerical solution techniques and how the modeling and simulation addresses biological variability.
- Sensitivity analysis is useful for establishing the degree to which the uncertainty in the model output(s) can be attributed to different sources of uncertainty in the model inputs [45, 46].

Evaluation metrics and test cases that can appropriately demonstrate the predictive performance of the modeling and simulation within the stated context of use should also be implemented and documented (Rule 6). Furthermore, as the modeling and simulation evolves and more data becomes available, the modeling and simulation should be continuously evaluated with increasing rigor. It is important to note that a commensurate increase in the evaluation rigor is expected as the expectations of Domain of Use, Use Capacity, and Strength of Influence of the context of use increase or change.

The challenge in evaluating and testing models and simulations, especially with regard to validation, often results from implementation- or discipline-specific considerations, such as with detecting overfitting in data-driven machine learning implementations [47] or in the validation of complex aggregate adverse outcome pathway models [16]. In the latter case, the approach to the model development implies the need for a hierarchical validation at each level of complexity, as well as a global validation of the model outcomes. Furthermore, the impact of validation depends on the model’s current and future intended utility, which may range from clinical decision-making to hypothesis generation in support of mental models. As a result, as the modeling and simulation evolves (e.g., matures in its life-cycle, alters its desired outputs) or more data becomes available, the modeling and simulation should be re-evaluated.

The engineering and physical science literature provides many excellent sources for finding techniques and processes associated with verification and validation [48, 49]. Each of the modeling cases in the additional files includes examples of evaluation activities, both qualitative and quantitative. A recent example illustrates the importance of including uncertainty quantification and sensitivity analysis techniques to increase our grasp of the predictive capability of a complex healthcare-oriented model system [50]. In this case, the authors applied traditional single value approaches, as well as independent and joint probability distribution Monte Carlo sampling to assess uncertainty propagation, via statistical analysis of output metrics; they also calculated Sobol indices to assess global sensitivity. As explained by the study authors, the lack of proper uncertainty quantification and sensitivity analysis limits the clinical application by denying the clinician (i.e., the decision maker) the robustness to tailor the tool to individual or intended patient populations. In effect, it gives the user of the model and simulation tools the decision-support insight that mirrors the formatted information familiar to them from a clinical trial cohort that addressed the range of variable parameters.

### Rule 4—list limitations explicitly

Biomedical phenomena can be convoluted at multiple scales, involving linkages across spatial and temporal scales and measurements made in vitro and in vivo in multiple species. To be tractable, modeling and simulation in healthcare must therefore make assumptions that are application-specific, which limits generalizability. As a result, modelers and developers must clearly identify the conditions under which their modeling and simulation ***cannot*** be relied upon and provide the rationale behind their statements. This not only lends credibility to the work, but also facilitates reuse by enabling individuals to assess whether the modeling and simulation is suitable for an alternate application. In fact, limitations could also be thought of as opportunities for future improvement and highlight paths to enhance the modeling and simulation.

Clear communications of potential aleatory and epistemic uncertainty is achieved by reporting the underlying limitations and assumptions of the model and simulation, most notably by putting into context the abstraction to the real-world system. This involves describing feature inclusion and exclusion, design decisions, types of outputs, and the relation of those outputs to the permissible uses of the model [36]. There can be overlap between this rule and other rules, such as defining the context clearly (Rule 1) and using appropriate data (Rule 2). Information provided under these other rules could be used to infer limitations. Rule 4, however, requires an explicit statement of the limitations, which can go beyond the obvious scope described by the other rules.

An example is the musculoskeletal model from Rajagopal, et al. [39] (see Additional file 1: Example 2). The model was shown to be suitable for simulating normal gait based on data from cadaver specimens and young healthy subjects (information captured under Rules 1 and 2). A logical limitation derived from such information is that the model is only suitable for simulating the stated conditions: normal gait in healthy individuals. Rajagopal and colleagues, however, provide an extensive list of limitations that go beyond these conclusions, demonstrating the need to explicitly describe the limits of each study. For example, they describe limitations in the model’s ability to estimate muscle force-generating capacity due to model simplifications.

The Rajagopal et al. [39] example also demonstrates how limitations can arise from a variety of sources. Exclusion of components in a model (e.g., cellular types, anatomy, signaling pathways), model simplifications (e.g., modeling a component in 2-D vs. 3-D), choice of parameter values (e.g., using data from one animal model for another), and other decisions made during development will all influence what can and cannot be modeled. Computational constraints and the validation protocol can introduce additional limitations. To provide a comprehensive description of the modeling and simulation limitations, it can be helpful to consider different audiences (e.g., limitations that are important to manuscript reviewers versus another modeler who wishes to extend the modeling and simulation framework) and different experimental scenarios (e.g., simulating varying gait speeds or pathologic conditions). Consideration of competing model implementations (Rule 9) may also expose modeling and simulation assumptions and limitations inherent to the numerical approach underlying the predictions (e.g., the use of forward versus inverse dynamics formulations for gait analysis). Taken together, such information provides a richer understanding of the modeling and simulation activity and therefore enhances its credibility.

### Rule 5—use version control

This rule refers to the need for version control for all model, software, data, and documentation files. Version control is a system for managing different iterations, or versions, of an asset or set of assets. Users sometimes start by using ad hoc approaches such as simple journaling (e.g., a laboratory diary) or periodic snapshots of the work in progress, possibly using filenames that include the timestamp to document model development and label different versions of models and data. More comprehensive approaches extend this functionality to also allow for 1) tracking changes between versions, 2) associating specific modifications to the creator, 3) and including annotations/comments/notes with each version. Such systems are known to greatly streamline tracking revisions to source code and documentation by automating the acquisition of version control information and history with higher frequency. Modern version control systems, notably Git [51] and Mercurial (hg) [52], are widely used examples. They work at the level of a set of files, rather than a single file, making it easy to commit (save as a version), log (list versions), clone (share), and diff (compare changes) for entire projects. Modern version control systems also facilitate collaboration on a particular project by identifying which individual makes a specific change and allowing individuals to work in parallel.

It is important to note that while most modelers and software developers follow these practices for building new models and software tools, it is less common that they use the same approach for documentation and simulation runs or to trace data. Having the ability to associate the data, documentation, and simulation logs to the specific version of the model is critical for accurate interpretation, repeatability, reproducibility, and debugging of the simulation predictions. This approach captures the history of the whole modeling and simulation life-cycle and, furthermore, it allows for traceability of model parameters and constants, thus ensuring complete reproducibility of the individual simulation runs, even by a third party (Rule 8). Depending on the discipline and intended context of use, version control may also relate to standardized practices of software quality assurance such as those of the IEEE [53]. For example, Neymotin et al. [54], in their modeling and simulation practice (see Additional file 1: Example 3), utilized the Mercurial version control management tool. They leveraged this not only for their modeling and simulation code but also for manuscripts and figures. This exemplifies the advantage of versioning through the whole modeling and simulation life-cycle: to establish provenance for data and to associate simulation outcomes to model versions.

### Rule 6—document appropriately

We define “document appropriately” to mean providing the range of information needed for others to (1) assess the credibility of the modeling and simulation activity both under the originally intended context as well as under new contexts and (2) understand the nuances of reproducing and using/reusing the associated code and model. Guidance for the comprehensive reporting of computational model studies is available in specific disciplines and for certain modeling techniques, e.g., for finite element analysis studies in biomechanics [55]. Journal publications can also provide some critical details about modeling and simulation activity, including information related to several of the Rules listed here, e.g., define context clearly (Rule 1). Due to the format and purpose, however, scholarly publications cannot comprehensively provide all the necessary information to describe the modeling and simulation. If the associated code or model is made available, as recommended when disseminating broadly (Rule 7), comments should be included in those files to explain implementation decisions and aid in their reuse. Additional documentation, such as a user or developer guide (see [56] for an example), can similarly provide detailed explanations not suitable for a journal publication. Useful information that might be found in such guides include best practice workflows for using the code or model, guidance on parameter selection, and common pitfalls.

The modeling and simulation activities related to work by Pennline and Mulugeta [57] illustrate how documentation is a continuous activity throughout the life-cycle of the model, and in their case, it directly targets informing the stakeholders. Not only were the code and interfaces documented, model features and credibility assessment have been routinely conveyed through reports, presentations, and briefings along with scholarly work. All these were curated for access by interested parties (see details in Additional file 1: Example 1).

### Rule 7—disseminate broadly

Traditional scientific dissemination involves publication with an emphasis on providing a thorough *Materials and Methods* section that permits others to replicate the experiments performed. Studies leveraging modeling and simulation generate and utilize many assets, including data, workflows, models, simulation software, and simulation results (raw and post-processed). “Disseminate broadly” refers not just to the traditional sharing of knowledge via publications, but also to the sharing of modeling and simulation assets.

When shared, these assets provide interested parties the opportunity to develop direct outcomes and/or by-products of the modeling and simulation. For example, data can be used to redevelop a model from scratch; workflows can be used to evaluate the completeness and reproducibility potential of the modeling and simulation processes; models and simulation software can be modified for new analyses with different context of uses; simulation results can also serve as a reference for conclusions made and support further in-depth analysis by a third party. The *Methods* section of traditional publication platforms are generally used to point towards data, document workflows, and describe the modeling and simulation software. Unfortunately, scholarly publication is generally not sufficient to embed all the details, and in the majority of cases, it is not even practical. Contemporary simulation studies and related models are often a combination of large pieces of software, sometimes instantiated in still larger specialized simulation software environments. And source code is now typically too large for printed listings. Even models that can be described succinctly as sets of equations are still not generally fully replicable due to different preferences of developers in choosing solver settings, e.g., integrators, randomizers. More detailed information on these issues can be found in domain-specific discussions of sharing computational models and related resources, e.g., in the discipline of biomechanics [58].

Examples of extensive sharing of modeling and simulation assets exist. Rajagopal et al. [39], publicly disseminated their musculoskeletal model, data to drive simulations, and documentation at SimTK [59] as part of their practice (Additional file 1: Example 2). Similarly, Neymotin et al. [54] leveraged a public repository [60] to share model code and published in open access journals (Additional file 1: Example 3). There may be a desire to limit the extent of dissemination. In such cases, sharing of models (and related data and documents) with a limited number of parties may still provide the benefit of third-party inspection and evaluation, which can enhance the credibility potential of the practice. For example, Pennline and Mulugeta [57] made their model available to its specific user base, in this case NASA researchers (see Additional file 1: Example 1). In other cases, the model was made available to reviewers (also the case for the study by Verma et al. [61] in Additional file 1: Example 4). Such strategies can support commercialization of the modeling and simulation practice while accommodating activities that have direct relevance to credibility.

We should note that dissemination of simulation software both in binary and source code has become a routine strategy in many subdisciplines of the biomedical research ecosystem. Sharing of models in machine and human readable format (in source markups) has also gained traction with varying degrees of success, depending on the biomedical domain [39, 62]. We recommend the use of existing repositories for disseminating code and models. While shared code and models may be placed on a laboratory website, it is preferable to utilize an archival location such as GitHub [63], journal websites, or specialized model databases, domain repositories and or general repositories such as [59, 60, 64–66] to ensure long-term availability of the shared assets. It would also be valuable to have links from such repositories to repositories that track related experimental and clinical data. To ensure discoverability, digital object identifiers should be acquired for the shared assets, which has become an available feature in many repositories, e.g., SimTK, figshare, Zenodo [59, 65, 66]. Ideally, it should be possible to reproduce one or more individual figures from a journal article using the downloaded code or model. As noted under other rules, code and accompanying documentation should include metadata relating to parameter provenance, simulation scenarios, extensibility and limits.

### Rule 8—get independent reviews

Following the other rules described in this article will significantly increase the credibility of a modeling and simulation activity. Having non-partisan third-party reviewers evaluate the activity will further enhance the community’s trust. For this rule, “third-party” reviewers refers to end-users or modelers/developers evaluating the activity in its entirety. Peer reviews of manuscripts, which include descriptions of the modeling and simulation activity, are discouraged as the sole form of third-party review since they provide a limited assessment, potentially only superficially addressing Rules 1–4. Choosing who reviews a modeling and simulation activity depends on the intended use and should be considered at the outset of a modeling and simulation activity.

For many modeling and simulation activities, a natural choice for a third-party reviewer would be the intended end-user. This might be a clinician, an educator, or a non-computationally inclined researcher. These individuals provide valuable feedback on the usability and relevance of the activity for their applications. Examples include a cardiologist assessing a model’s suitability for determining patient-specific pharmacological treatment for pulmonary hypertension or a health department policy maker evaluating a population model for deciding the most effective communication campaign. Specific questions these end-user reviewers want answered often relate to the other Rules:

- Define context clearly (Rule 1) and List limitations explicitly (Rule 4): End-user reviewers need sufficient information to evaluate whether or not the activity is suitable for their research question of interest.
- Use appropriate data (Rule 2) and Evaluate within context (Rule 3): End-user reviewers want to examine the validation evidence to decide the level of trust they have in the model output(s).
- Document appropriately (Rule 6): Clearly written documentation with sufficient details will enable a reviewer to answer questions that can arise when reproducing a modeling and simulation study or adapting a tool to their application.
- Test competing implementations (Rule 9): The end-user reviewer may be interested in how results of the modeling and simulation activity compares with those generated by existing tools or implementations, as these provide a previously established reference of performance.
- Conform to standards (Rule 10): If a community has common formats or methods for any modeling and simulation activity, including its evaluation, the end-user reviewer will be interested in how the modeling and simulation activity follows these standards. Conforming to such standards facilitates comparisons and enhances interactivity with the community’s other modeling and simulation activities.

Modelers/developers assess a modeling and simulation activity from a development perspective, wanting to extend the modeling and simulation activity for a novel use or compare it with other similar activities. So their questions necessarily differ from those of end-user reviewers, though they can still be categorized into the stated Rules. For example, sufficient documentation for an end-user reviewer may consist simply of the mechanics of running a model, while a modeler reviewer would want details of how the model was constructed, the parameters used in running the model, and references for extending the simulation code. Additionally, a modeler reviewer will often want direct access to the source code, models, and underlying data, and thus using version control (Rule 5) and disseminating broadly (Rule 7) take on increased importance for these reviewers.

Strategies to involve peer-reviewers of scholarly publication to also perform third-party review of model and modeling and simulation processes have been tested in biomedical disciplines, e.g., for computational biomechanics [67]. The musculoskeletal model described by Rajagopal et al. [39] was disseminated during the submission of their manuscript (also see notes in Additional file 1: Example 2). The reviewers performed simulations to reproduce reported results. This exercise identified problems with input data that were utilized in the original submission and resulted in the acquisition of higher quality data by the study authors for the resubmission. Incorporating third-party review, not only of publications but also of models, demonstrated the importance of dissemination (Rule 7) to facilitate a comprehensive review. While both the reviewers and the practitioners of the modeling and simulation workflow noted the added burden on the overall review process, they also strongly agreed that the quality of modeling and simulation increased after such reviews.

One outstanding question is how to identify and engage non-partisan reviewers. No best practices have been developed in this regard. Recent funding initiatives, e.g., from the Interagency Modeling and Analysis Group and the Multiscale Modeling Consortium [30], ask grantees proposing computational modeling studies to provide plans for addressing and reporting modeling and simulation credibility. Ideas worth exploring include budgeting a portion of grant funding to hiring third-party reviewers to the activity of establishing collaborations with other grantee labs to assess each other’s models. Verma et al. [61] relied on re-implementations by an independent laboratory member who was not involved with the project (see Additional file 1: Example 4). The model by Neymotin et al. [54] was reviewed for run-capability on multiple platforms by a ModelDB [60] curator (see Additional file 1: Example 3). Note that the ideal cross-lab validation involves groups that are not working or have not previously worked together. Publishers may provide more comprehensive reviews of modeling and simulation studies in the future as well. The journal *PLOS Computational Biology* recently launched a pilot to provide simulation and results verification for authors using the Center for Reproducible Biomedical Modeling’s [68] services. Ultimately, which third-party review mechanism is utilized is not nearly as critical as having thoughtful, impartial evaluations predicated on accepted guidelines/requirements, which improve not just the credibility of the modeling and simulation activity but also the activity itself.

### Rule 9—test competing implementations

Model and simulation development often results from the effort to improve an existing model or analysis process by incorporating new techniques or knowledge. Thus, comparison of a competing model application to a prior implementation can provide insight on the evolution of the model strategies and algorithms, as well as on the impact of results from which historical conclusions have been drawn. For the healthcare model practitioner, this comparison provides valuable understanding into the model behavior with respect to familiar standards of performance. A comprehensive contrast of modeling strategies also informs the user about the interplay between model algorithms, operational factors, and model parameters in supporting decision tools and strategies across a range of application scenarios. Differences may also illustrate where future model improvements can be made or clarify that decisions should be supported by an ensemble of competing model output(s). When combined with uncertainty quantification approaches in Rule 3, this competitive comparison of modeling techniques provides the model practitioner with much needed insight for projecting how a model can support their specific implementation and may lead to ensemble application of competing models to overcome individual limitations.

In the case of a unique model development effort, where the model developer and practitioner lack competing implementations, similar insight may be drawn from pursuing alternative formulations or numerical implementations. The conceptual modeling phase of modeling and simulation often involves weighing the pros and cons of competing approaches, and thus, the decision to use a particular approach may provide a valuable understanding of modeling and simulation performance. In practice, this may be achieved by implementation on alternative platforms or in alternative programming languages that may require different orders of operations and can illustrate important features of model performance. Reporting such implementation tests establishes due diligence in the practical application of a model.

The benefits to this credible practice are exemplified by successful application in the physical science and engineering disciplines, where the use of so-called surrogate models extends testability [36]. These surrogate models, notably data-driven models now extending to machine learning, provide a continuous comparative representation at a focal area of application, usually without consideration of all the original model and simulation underlying limitations. In this case, the intent is to enhance the testability of the model in some combined parameter space that may not be directly measurable. The surrogate models would also need to follow credible practice to allow evaluation as viable comparators. In relevance to this rule for healthcare, the modeling and simulation practitioners may decide to implement different simulation strategies or use different models for the same purpose. In modeling and simulating musculoskeletal movements, Rajagopal et al. [39] decided to use two other publicly available and commonly used musculoskeletal models, specifically to assess relative computational cost (see Additional file 1: Example 2). The implementation of different models or different simulation strategies may be challenging or burdensome. Hence, efforts analyzed in Additional file 1: (Examples 3 and 4; Neymotin et al. and Verma et al. [54, 61], respectively) did not attempt any other implementations. Nonetheless, explicit acknowledgment of the lack of such attempts can provide the audience a roadmap for the development of alternative strategies.

The burden of testing and comparing competing implementations need not fall on a single group, however. Competing implementations by different modeling and simulation teams can be curated through “grand challenge competitions”, e.g., similar to the one conducted for the prediction of in vivo knee joint loading [69]. Organic collaborations among teams with synergistic interest in a specific modeling goal can also serve as a framework for comparing modeling and simulation for the same context of use but with different flavors of implementation [70].

### Rule 10—conform to standards

Just as in the case of designing, implementing and reporting rigorous and repeatable experimental protocols, user communities expect model development and utilization to conform to applicable, and sometimes discipline-specific, development guidelines, operating procedures, and standards. Paraphrasing the International Organization for Standardization [71], standards, when consistently applied, represent a means of providing requirements, specifications, and guidelines that establish that the modeling and simulation materials and products fit the intended purpose (e.g., modeling and simulation is appropriate for the context of use). The collection of relevant standards represents a minimum set of guidelines consolidating the applicable community expectations. Failing to follow and report outcomes associated with applicable community guidelines reduces confidence in the modeling and simulation and increases the difficulty of communicating credibility. In contrast, following pertinent standards and practices conforms with expectations and therefore promotes acceptance and utilization.

The decision of which standards (coordinated or de facto) to follow depends on the discipline, the institution leading the development, as well as the standards expected by the user community and any governmental or private regulating bodies. The importance of specific standards will vary with the development stage of the modeling and simulation application. The Ten Rules can provide an overarching framework for considering which standards to incorporate into a modeling and simulation project. One might expect to follow standards from institutional review boards for data acquisition and/or the use of animal and human subjects (Rule 2), standards and guidelines related to verification, validation, and uncertainty quantification [25, 43, 44, 53, 72–74] (Rule 3), and/or community-accepted best practices for dissemination, such as using common markup languages [75] (Rule 7). Many institutions require reporting modeling and simulation products by following in-house or discipline-specific minimum reporting standards (Rule 6) [55]. We encourage adherence to standards which promote transparency, i.e., open-source technology, to improve insight into and adoption of modeling and simulation whenever possible. The Internet protocols is an example of such open standards. More information about open standards is available at [76].

A few examples of adopting well-defined standards or de facto best practices (for overall workflows, model representation, or individual processes) are worth noting. The whole workflow of Pennline and Mulugeta [57] (see Additional file 1: Example 1) was faithful to NASA-STD-7009, a technical standard that establishes uniform modeling and simulation practices related to NASA’s mission [6]. Verification and validation efforts of Rajagopal et al. [39] (see Additional file 1: Example 2) hinged on guidance on best practices applicable to musculoskeletal modeling [72]. Similarly, modeling by Neymotin et al. [54] (see Additional file 1: Example 3) relied on best practices encouraged in training sessions of NEURON [77], with model dissemination conforming to the standards of ModelDB [60]. Both of these are geared towards simulation of neurons. Verma et al. [61] represented their model in Systems Biology Markup Language [78] (see Additional file 1: Example 4), an open standard for the exchange of computational models in system biology.

Clearly, the application of appropriate standards challenges the developer to identify and follow these standards early in the development cycle. This is especially true as modeling and simulation in healthcare increasingly requires a multidisciplinary approach to address modeling challenges to engage in higher stakes questions. Clear and on-going developer-user communication is needed to effectively combine multidisciplinary standard formats, methods of evaluation, and development requirements. The benefits of aligning a modeling and simulation activity with existing standards are worthwhile, improving community perception of the model application and fostering a deeper understanding of the development rigor of the modeling and simulation product.

## Conclusions: scope and utility of rules for credible practice of modeling and simulation

The biomedical sciences and clinical disciplines are diverse and multidisciplinary, and the modeling and simulation community is highly heterogeneous from domain experts to novice enthusiasts. Intentions of modeling and simulation vary dramatically as well, ranging from medical training to hypothesis generation to clinical decision-making. The perception and relative importance of the Ten Rules described here and the intensity at which they are applied are expected to be influenced by multidisciplinary, organizational, and contextual factors. The contribution of this study hinges on treating credibility as a term inclusive of validation but incorporating many other aspects that critically impact the overall quality of the modeling and simulation practice. Such a comprehensive treatment is rare in literature, nonetheless exists as a sign towards the need for such guidance, e.g. Law, Rabeau, Behrend, and Bodner et al. [19–21, 24]. We accomplish this by establishing a set of expected activities and information that is sufficient to establish credibility for the user/decision maker’s unique use case, understanding that available models will be used by both intended and unforeseen users. The rules are intended to be applicable throughout the entire model life-cycle, which can include stages for ideation, development, calibration, benchmarking, accreditation, use as originally intended, and reuse. The rules are not limited solely to model development and testability. A majority of activities in relation to the Ten Rules span the whole modeling and simulation life-cycle. A few focus on certain stages, i.e., evaluation activities primarily belong to benchmarking. A more detailed correspondence between the Ten Rules and modeling and simulation life-cycle stages is provided as (Additional file 2: Table SM-3). The relationship between the rules and modeling stages demonstrates the comprehensiveness of the Ten Rules that extend beyond singular activities.

As a tightly linked cohort, they represent a customizable framework to tailor to the domain of application, state of development, and stakeholders. Therefore, these rules serve as a reference to guide the everyday practice and communication of modeling and simulation credibility for developers, researchers, and healthcare stakeholders in the utilization of modeling and simulation products. Followed in their entirety, these Ten Rules support the execution and communication of activities that realize the metascience themes of reproducibility, comprehensibility, and transparency. They also reinforce the acceptance of a modeling and simulation credible practice whose foundation is premised on validation and other testable measures.

To enhance the practitioners’ understanding of the potential virtues of the Ten Rules credible practice, a comprehensive perspective on the benefits of including and the pitfalls of excluding activities related to each rule can be found in the Additional file 2: Table SM-4. When following these practices to enhance the communication of model and simulation credibility, we recommend that the practitioner state if a rule is implemented or not. If it is implemented, descriptions of the implementation describing how and the degree of compliance with the rule relative to the model’s context of use should be provided. Similarly, if a rule is not implemented, details explaining the decision describing why should be given. The implications of not addressing individual rules should indicate the potential impacts on the ability to support scientific or clinical decision and policy development, all in relevance to the model’s context of use. An example of this would be that an inability to complete Rule 8 (Get Independent Reviews). One impact could be the lack of independent affirmation of the influence of the assumptions of limitations, thus influencing model and simulation interpretation, given in Rule 4. By giving the user such transparent and comprehensive insight into the model and simulation’s development life-cycle, the modeler enables the user community to critically evaluate the level of confidence in the model’s intended use and the feasibility of the model’s application in future implementations.

It is highly likely that holistic practices for credible modeling and simulation will be equally applicable to emerging data-driven computational strategies for scientific discovery and clinical care, such as big data analytics and machine learning. In order to conform to these rules, we anticipate the development of tools as well as infrastructure to customize or automate potentially burdensome activities. We also expect and have already observed the expansion and evolution of these rules as well as their application, as achieving a good representation of credibility is a continuous process. As such, we have adopted an ad hoc, but iterative process for updating the rules and supporting guidelines based on the feedback we receive from the research community (Fig. 2). Users can now complete an online form that can be continuously updated on the Interagency Modeling and Analysis Group wiki site [9]. We plan to formalize the process, which may include evolving the Committee into an independent body that will oversee the perpetuation of the rules and guidelines beyond the Interagency Modeling and Analysis group and the Multiscale Modeling Consortium initiative.

Few things serve to educate and exemplify these Ten Rules for credible practice of modeling and simulation better than examples of the modeling and simulation community following and communicating these credible practices. As part of our effort to promote modeling and simulation credible practices, the Committee has created an open repository of models and simulations that epitomize application of each of the Ten Rules. Updated yearly, this repository [79], hosts modeling and simulation links, descriptions, and summaries of example credibility descriptions, as well as links to common standards, modeling and simulation repositories, and suggested reporting formats for modeling and simulation credibility. Since its formation, the Committee has followed an agile, continuous process to understand modeling and simulation activities in light of their impact on perceived credibility of the practice. A diverse group of stakeholders have contributed to this understanding through discussions, position statements, surveys, and so on, and continue to do so. The Committee’s efforts have been inclusive, essentially open to anyone with the dedication to contribute. The transparency of our activities provides insight into our collective thought process through openly accessible meeting minutes, discussion summaries, survey data, presentations, and implementation examples. The Committee routinely solicits and incorporates feedback from the Interagency Modeling and Analysis group and the Multiscale Modeling Consortium [9], and grantee activities continue to integrate the Ten Rules as part of their modeling and simulation practices via federal funding programs. This portrayal is intended to demonstrate that the Committee practices what it preaches, supporting FAIR principles and mirroring the philosophy of the Ten Rules. The Committee welcomes suggestions, example submissions, and comments on credible practice activities to share with the broader modeling and simulation healthcare community, with the hopes of fostering more in-depth discussion and commonplace adoption of these guidelines in the credible practice of modeling and simulation in healthcare.

## Supplementary information

**Additional file 1:** Supplementary Examples: A collection of 4 published studies in different biomedical disciplines illustrating high level correspondence between modeling and simulation activities and the ten rules for credible practice of modeling and simulation in healthcare.

**Additional file 2:** Supplementary Tables: A collection of tables intended to provide the interested reader additional insight into the ten rules’ development details and the benefit of applying the ten rules throughout a model and simulation life cycle.

## Data Availability

Extensive documentation of the methodologies, progression and results of this multi-year study are publically available on the Committee’s Wiki page.^1^ We have also published the raw data for the global stakeholder survey study with a DOI for public use.^2^

## Abbreviations

FAIR: Findable, Accessible, Interoperable, Reusable
FDA: Food and Drug Administration
NIH: U.S. National Institutes of Health
