# Supplementary material for: Credible practice of modeling and simulation in healthcare: ten rules from a multidisciplinary perspective
Source: J Transl Med. 2020 Sep 29;18:369. doi: 10.1186/s12967-020-02540-4 (PMC7526418; doi:10.1186/s12967-020-02540-4)
Supplement: Supplementary file 1 — Additional file 1: Supplementary Examples: A collection of 4 published studies in different biomedical disciplines illustrating high level correspondence between modeling and simulation activities and the ten rules for credible practice of modeling and simulation in healthcare. [file 12967_2020_2540_MOESM1_ESM.docx]

# Supplementary Material - Examples

This document is prepared as supplementary material for the publication titled “Credible practice of modeling and simulation in healthcare: ten rules from a multidisciplinary perspective” [1].

Modeling and simulation activities of a collection of published studies in different biomedical areas were analyzed. The analyses aimed for a high level understanding of the correspondence between modeling and simulation activities and ten rules for credible practice of modeling and simulation in healthcare [1]. The analyses included additional self-reported information provided by the modelers of said studies. These studies, including their modeling areas of interest, are:

1. **Study:** A computational model for simulating spaceflight induced bone remodeling [2].

**Modeling Area:** First-order nonlinear differential equation-based modeling of bone changes.

1. **Study:** Full-body musculoskeletal model for muscle-driven simulation of human gait [3].

**Modeling Area:** Rigid-body lower-limb musculoskeletal modeling and simulation.

1. **Study:** Multitarget multiscale simulation for pharmacological treatment of dystonia in motor cortex [4].

**Modeling Area:** Network simulations of compartmental cell models of the motor cortex.

1. **Study:** Modeling the dynamics of human liver failure post liver resection [5].

**Modeling Area:** Ordinary differential equation-based modeling of liver regeneration.

A living version of a collection of these examples can be found online [6].

## References

1. Erdemir A, Mulugeta L, Ku JP, Drach A, Myers JG, Horner M, Morrison T, Vadigepalli R, Lytton WW. Credible practice of modeling and simulation in healthcare: ten rules from a multidisciplinary perspective. J Transl Med.
2. [Pennline JA, Mulugeta L. A computational model for simulating spaceflight induced bone remodeling.](http://paperpile.com/b/KXjwuZ/BqRz) The 44th International Conference on Environmental Systems. July 13-17, 2014, Tuscon, Arizona. uri: <https://ttu-ir.tdl.org/handle/2346/59750>.
3. Rajagopal A, Dembia CL, DeMers MS, Delp DD, Hicks JL, Delp SL. Full-body musculoskeletal model for muscle-driven simulation of human gait. IEEE Trans Biomed Eng. 2016 Oct;63(10):2068-79. doi: 10.1109/TBME.2016.2586891.
4. Neymotin SA, Dura-Bernal S, Lakatos P, Sanger TD, Lytton WW. Multitarget multiscale simulation for pharmacological treatment of dystonia in motor cortex. Front Pharmacol. 2016 Jun 14;7:157. doi: 10.3389/fphar.2016.00157.
5. Verma BK, Subramaniam P, Vadigepalli R. Modeling the dynamics of human liver failure post liver resection. Processes (Basel). 2018 Aug;6(8):115. doi: 10.3390/pr6080115.
6. Ten Simple Rules Examples - cpms [[Internet]. [cited 2020 Jul 25]. Available from:](http://paperpile.com/b/KXjwuZ/O1dK) <https://simtk.org/plugins/moinmoin/cpms/Ten%20Simple%20Rules%20Examples>

#

# Supplementary Material - Example 1

This document is prepared as supplementary material for the publication titled “Credible practice of modeling and simulation in healthcare: ten rules from a multidisciplinary perspective” [1].

## Study

A computational model for simulating spaceflight induced bone remodeling [2].

## Modeling Area

First-order nonlinear differential equation-based modeling of bone changes.

## Background

A finite element model was developed to understand bone remodeling and demineralization mechanisms in microgravity in order to: 1) appropriately quantify long term bone health risks (osteoporosis & bone fracture), and 2) establish appropriate countermeasures.

## Analysis History

A living version of this example can be found online [3].

- Original review by Lealem Mulugeta (study co-author), May 16, 2018. Additional insight was provided based on knowledge of modeling and simulation activities beyond those reported as part of the study publication.
- Updated by Lealem Mulugeta (study co-author), May 10, 2020.
- Edited and formatted by Ahmet Erdemir on July 25, 2020 as supplementary material for related publication [1].

## Disclaimer

THIS EXAMPLE DOES NOT NECESSARILY DEMONSTRATE THE LEVEL OF DETAIL REQUIRED TO FULLY SATISFY THE COMMITTEE’S CREDIBILITY GUIDELINES [1]. THE GRANULARITY OF HOW EACH RULE IS APPLIED IS SOLELY DEPENDENT ON THE NEEDS OF EACH MODELING AND SIMULATION PROJECT.

**Rule 1 – Define context clearly**

- Domain of Use: Translational research. The bone model is being developed primarily as a spaceflight research tool, and not as a clinical tool.
- Use Capacity: The model is intended to provide additional data to gain insight on (1) mechanisms of bone demineralization in microgravity, and (2) the volumetric changes in response to in-flight and post-flight exercise countermeasures.
- Strength of Influence: This version of the model only targets the femoral neck to illustrate the capacity to make effective predictions. The model is not being developed to predict bone fracture.

**Rule 2 – Use contextually appropriate data**

- Model development data: Since bone parameter values are still under active research by the scientific community, average values from the scientific literature were used. Parameters that were utilized in the model include: resorption depth (depth of modeling unit) for trabecular hemi-osteo; resorption depth for cortical bone; activation frequency; TG-beta 1.
- Validation data came from a regression "sub-model," which was developed using total femur DEXA aBMD and QCT vBMD [BMD = bone mineral density] data from the flight study reported in Lang et al. [4] – raw data was provided by NASA’s Life Science Data Archives [5]. This regression “sub model” helped expand the data set to draw on to validate the computational model, as well as run investigative simulations.

**Rule 3 – Evaluate within context**

- Validated model's ability to reproduce the observed behavior under consideration (e.g., bone mineral density (BMD) or bone volume fraction (BVF) changes) in comparison to an appropriate referent, specifically:
  - Bone Volume Fraction (BVF) compared against 3 other model predictions and against experimental data from Tsangari et al. [6].
  - Volumetric BMD (vBMD) predicted by model compared against that from quantitative computed tomography (QCT) for both trabecular and cortical bone for 4 subjects after 70 days of bed rest (spaceflight analog).
  - Time course of Areal BMD (aBMD) predicted by model versus that from dual-energy X-Ray absorptiometry (DEXA) for 18 subjects during 17 weeks of bed rest (spaceflight analog) (LeBlanc et al. [7]). Raw data were provided by NASA’s Life Science Data Archives [5] and NASA's Bone Research Lab.
- Rigorous verification, sensitivity and uncertainty analysis of the system of equations, parameters and variables were identified as future work.

**Rule 4 – List limitations explicitly**

- Limitations in the modeling approach include:
  - Remodeling formulation is limited to porosity, thus restricting it to density changes within the trabecular region and to intracortical density changes. It does not cover periosteal apposition or endocortical change. Geometry changes in the bone site are not modeled.
  - The model does not include the effects of sclerostin, calcitonin, osteopontin, or interleukins, some of which may play a role bone loss in microgravity and with disuse in 1g.
  - Preliminary validation analysis of the computational predictions for deconditioning has only been done for up to 4 months in duration.
  - The validation data used are from bed rest control subjects as an analog to gravitational unloading due to exposure to microgravity.
  - The computational model is best suited for the mature adult between 25 and 55 years of age (typical age of an astronaut). Capability to make subject specific predictions is limited. Age and gender differences are not yet factored in when initializing model variables.
- Limitations imposed by the state of knowledge in bone science:
  - There is a degree of uncertainty and variation in remodeling unit geometry and dimensions reported in the literature.
  - It is difficult to guarantee that the remodeling unit values used in the model agree for the particular skeletal site of interest.
  - There is uncertainty in the way ash fraction is modeled, and the full potential range of values estimated from experimental studies is not completely understood.
  - Activation frequency and activation density are inherently difficult to appropriately model due to the lack of human values at skeletal sites other than the iliac crest or rib.
  - There are several potential algebraic schemes for mapping initial data values to model state variables. They depend on several possible definitions of ash fraction and how the steady state version of their respective equations are used.

**Rule 5 – Use version control**

- Regular commits are made to a Subversion repository.
- Stable version releases are created with appropriate documentation.

**Rule 6 – Document appropriately**

- Code was documented sufficiently for modelers and scientists.
- Graphical user interface was developed for intuitive use by end users.
- Every model delivery to stakeholders was accompanied with a report summarizing model features and credibility, see Pennline and Mulugeta [8] for an example.
- Presentations and briefings provided to stakeholder community at quarterly meetings, annual agency reports, and annual HRP Investigators’ Workshop.
- Peer-reviewed articles, conference presentations and technical memos were produced regularly (search Pennline and Mulugeta at the NASA Technical Report Server [9]).

**Rule 7 – Disseminate broadly**

- The code was developed and made available for use by NASA researchers. It was not intended for release to the general public.
- Peer-reviewed articles and conference presentations are available for public consumption (search Pennline and Mulugeta at the NASA Technical Report Server [9]).

**Rule 8 – Get independent reviews**

- In accordance to NASA STD 7009 [10], technical reviews were conducted to ensure critique from key stakeholders (see an online example [11]).
- In addition to obtaining feedback from the key stakeholders, NASA’s Research and Clinical Advisory Panel (external subject matter experts) were provided a summary report. The Panel used this report to provide feedback to the NASA Bone Discipline Lead regarding the potential utility and weakness of this model with respect to its context of use.

**Rule 9 – Test competing implementations**

- This is an ongoing process. The foundational model was formed by comparing, contrasting, combining, and modifying previously developed set of biochemical, cellular dynamics, and mechanical stimulus equations in the literature.

**Rule 10 – Conform to standards**

- The model and simulations were developed and applied in accordance to NASA’s Standard for Models and Simulations (NASA STD 7009) [10].
- All human subject data were used in accordance to HIPAA.

## References

1. Erdemir A, Mulugeta L, Ku JP, Drach A, Myers JG, Horner M, Morrison T, Vadigepalli R, Lytton WW. Credible practice of modeling and simulation in healthcare: ten rules from a multidisciplinary perspective. J Transl Med.
2. [Pennline JA, Mulugeta L. A computational model for simulating spaceflight induced bone remodeling.](http://paperpile.com/b/KXjwuZ/BqRz) The 44th International Conference on Environmental Systems. July 13-17, 2014, Tuscon, Arizona. uri: <https://ttu-ir.tdl.org/handle/2346/59750>.
3. Pennline2014- cpms [[Internet]. [cited 2020 Jul 25]. Available from:](http://paperpile.com/b/KXjwuZ/O1dK) <https://simtk.org/plugins/moinmoin/cpms/Pennline2014>
4. Lang T, LeBlanc A, Evans H, Lu Y, Genant H, Yu A. Cortical and trabecular bone mineral loss from the spine and hip in long-duration spaceflight. J Bone Miner Res. 2004 Jun;19(6):1006-12. doi: 10.1359/JBMR.040307.
5. LSDA [[Internet]. [cited 2020 Jul 25]. Available from:](http://paperpile.com/b/KXjwuZ/O1dK) <https://lsda.jsc.nasa.gov/>
6. Tsangari H, Findlay DM, Fazzalari NL. Structural and remodeling indices in the cancellous bone of the proximal femur across adulthood. Bone. 2007 Jan;40(1):211-7. doi: 10.1016/j.bone.2006.07.007.
7. Leblanc AD , Schneider VS, Evans HJ, Engelbretson DA, Krebs JM. Bone mineral loss and recovery after 17 weeks of bed rest. J Bone Miner Res. 1990 Aug;5(8):843-50. doi: 10.1002/jbmr.5650050807.
8. Pennline J, Mulugeta L. The Digital Astronaut Project computational bone remodeling model (beta bersion) Bone Summit Summary report. November 1-5, 2013, Houston, Texas. url: <https://ntrs.nasa.gov/archive/nasa/casi.ntrs.nasa.gov/20140003236.pdf>
9. NASA Technical Reports Server (NTRS) - Basic Search [[Internet]. [cited 2020 Jul 25]. Available from:](http://paperpile.com/b/KXjwuZ/O1dK) <https://ntrs.nasa.gov/>
10. NASA STD 7009 - National Aeronautics and Space Administration. Standard for models and simulations. NASA; 2016 Jul. Report No.: NASA-STD-7009. url: <https://standards.nasa.gov/standard/nasa/nasa-std-7009>
11. Nelson E, Mulugeta L, Walton M, Myers J. How to develop and interpret credibility assessments of numerical models for human research: NASA-STD-7009 demystified. Human Research Program Investigators’ Workshop, February 12-13, 2014, Galveston, Texas. url: <https://ntrs.nasa.gov/archive/nasa/casi.ntrs.nasa.gov/20140017305.pdf>

Supplementary Material - Example 2

This document is prepared as supplementary material for the publication titled “Credible practice of modeling and simulation in healthcare: ten rules from a multidisciplinary perspective” [1].

## Study

Full-body musculoskeletal model for muscle-driven simulation of human gait [2].

## Modeling Area

Rigid-body lower-limb musculoskeletal modeling and simulation.

## Background

Musculoskeletal models provide a non-invasive means to study human movement and predict the effects of interventions on gait. This study describes the validation of an open-source 3-D musculoskeletal model with high-fidelity representations of the lower limb musculature of healthy young individuals that can be used to generate accurate simulations of gait.

## Analysis History

A living version of this example can be found online [3].

- Original analysis report by Lealem Mulugeta on March 20, 2018.
- Updated by Joy Ku on April 23, 2020. Added categories for defining context (Rule 1).
- Updated by Ahmet Erdemir on July 25, 2020. Added additional citations in regard to independent review (Rule 8) and conformance to standards (Rule 10).
- Edited and formatted by Ahmet Erdemir on July 25, 2020 as supplementary material for related publication [1].

## Disclaimer

THIS EXAMPLE DOES NOT NECESSARILY DEMONSTRATE THE LEVEL OF DETAIL REQUIRED TO FULLY SATISFY THE COMMITTEE’S CREDIBILITY GUIDELINES [1]. THE GRANULARITY OF HOW EACH RULE IS APPLIED IS SOLELY DEPENDENT ON THE NEEDS OF EACH MODELING AND SIMULATION PROJECT.

**Rule 1 – Define context clearly**

- Domain of Use: Fundamental research.
- Use Capacity: This is an open-source, three-dimensional musculoskeletal model that
  - has high-fidelity representations of the lower limb musculature of **healthy, young adult individuals**, and
  - is **computationally fast** enough for use in muscle-driven simulations.
- Strength of Influence: Is suitable for simulating **normal gait** - specifically for walking and jogging/running, but should be tested before use for activities that require high knee flexion such as sprinting and cycling.

**Rule 2 – Use contextually appropriate data**

- Data used to develop the model include 1) motion capture data from 41 retro-reflective markers tracked at 100 Hz using an eight-camera motion capture system; 2) ground reaction forces and moments measured at 2000 Hz using over-ground force plates; 3) musculotendon parameters derived from previous anatomical measurements of 21 cadaver specimens and magnetic resonance images of 24 young healthy subjects.
- Expected variability in the data is provided in Supplemental Table I of Rajagopal et al. [2].
- Data used to validate the model: experimental electromyography (EMG) data for the gluteus maximus, gluteus minimus, rectus femoris, vastus lateralis, biceps femoris long head, gastrocnemius lateralis, tibialis anterior, and soleus.

**Rule 3 – Evaluate within context**

- Tested model fidelity criteria by 1) qualitative comparison of musculoskeletal geometry of the model to experimental data; 2) quantitative and qualitative verification of simulated muscle-generated joint moments to inverse dynamics joint moments; 3) qualitative validation of simulated muscle activity to EMG data.
- Comprehensive testing and sensitivity analysis are still needed.
- Tested computational speed by comparing the speed of the Full Body Model to generate a single gait cycle simulation relative to other frequently used musculoskeletal models.

**Rule 4 – List limitations explicitly**

- Model does not contain representations of all lower limb muscles or representations of ligaments or other soft tissues.
- Chosen musculotendon parameters represent an average individual based on experimental data from literature, and these data contain variability, which is not captured in the model.
- It is assumed that as a muscle-tendon unit changes length, all fibers in the muscle change length equally.
- Model tested within defined ranges of motion: 40° plantarflexion to 30° dorsiflexion; 0° to 120° knee flexion; 30° hip extension to 120° hip flexion; 50° hip abduction to 30° hip adduction; and 40°hip external rotation to 40° hip internal hip rotation.
- Users should always test within the kinematic space the model will be used - particularly for movement with higher knee flexion angles.

**Rule 5 – Use version control**

- All versions of the model, associated data, and documentation are managed using the subversion repository provided online at the project website [4].

**Rule 6 – Document appropriately**

- All associated documentation (e.g., readme file) and publications are freely available for download from the project website at SimTK [4].

**Rule 7 – Disseminate broadly**

- The model, data used to create the simulations, documentation, and publications are freely available for download from the SimTK project site. The user community is also encouraged to make refinements and share them.

**Rule 8 – Get independent reviews**

- Prior to making the model publically available, the model was submitted for independent review in conjunction with publication review of their manuscript. During the initial submission of the manuscript and model,
  - all four reviewers were able to reproduce the results reported in the manuscript;
  - reviewers noted a need for more streamlined and documented process to re-run simulations; and
  - several data discrepancies were identified in the initial submission of the manuscript and model [5].
- With resubmission of the manuscript and model, Rajagopal et al. made several enhancements that improved the usability of the model and alleviated data discrepancies.

**Rule 9 – Test competing implementations**

- Simulation computation time of the model was compared to two other commonly used OpenSim models: the Delp model modified to include arms, and the Arnold model (both cited in Rajagopal et al. [2]).
- Model’s fidelity to simulate normal gait was not compared with these two commonly used models or any other model.

**Rule 10 – Conform to standards**

- Model testing conformed to published guidelines for best practices “Best practices for verification and validation of musculoskeletal models and simulations of human movement” [6].
- Human subject testing was carried out with Institutional Review Board approval.
- Data collection, processing, and reporting methods conformed to practices generally accepted by the biomechanics community.

## References

1. Erdemir A, Mulugeta L, Ku JP, Drach A, Myers JG, Horner M, Morrison T, Vadigepalli R, Lytton WW. Credible practice of modeling and simulation in healthcare: ten rules from a multidisciplinary perspective. J Transl Med.
2. Rajagopal A, Dembia CL, DeMers MS, Delp DD, Hicks JL, Delp SL. Full-body musculoskeletal model for muscle-driven simulation of human gait. IEEE Trans Biomed Eng. 2016 Oct;63(10):2068-79. doi: 10.1109/TBME.2016.2586891.
3. Rajagopal2016 - cpms [[Internet]. [cited 2020 Jul 25]. Available from:](http://paperpile.com/b/KXjwuZ/O1dK) <https://simtk.org/plugins/moinmoin/cpms/Rajagopal2016>
4. SimTK: Full Body Model for use in Dynamic Simulations of Human Gait: Project Home [Internet]. [[cited 2020 Jul 25]](http://paperpile.com/b/KXjwuZ/O1dK). Available from: <https://simtk.org/projects/full_body>
5. Erdemir A, Guess TM, Halloran JP, Modenese L, Reinbolt JA, Thelen GD, Umberger BR. Commentary on the integration of model sharing and reproducibility analysis to scholarly publishing workflow in computational biomechanics. IEEE Trans Biomed Eng. 2016 Oct;63(10):2080-2085. doi:10.1109/TBME.2016.2602760.
6. Hicks JL, Uchida TK, Seth A, Rajagopal A, Delp SL. Is my model good enough? Best practices for verification and validation of musculoskeletal models and simulations of movement. J Biomech Eng. 2015 Feb 1;137(2):020905. doi: 10.1115/1.4029304.

# Supplementary Material - Example 3

This document is prepared as supplementary material for the publication titled “Credible practice of modeling and simulation in healthcare: ten rules from a multidisciplinary perspective” [1].

## Study

Multitarget multiscale simulation for pharmacological treatment of dystonia in motor cortex [2].

## Modeling Area

Network simulations of compartmental cell models of the motor cortex.

## Background

This multiscale model of the primary motor cortex ranges from molecular, up to cellular, and network levels. It contains 1715 compartmental model neurons with multiple ion channels and intracellular molecular dynamics. The model wiring is based on electrophysiological data obtained from mouse motor cortex circuit mapping experiments. The model was used to reproduce patterns of heightened activity seen in dystonia by applying independent random variations in parameters to identify pathological parameter sets.

## Analysis History

A living version of this example can be found online [3].

- Original analysis report by William Lytton (study co-author), July 15, 2017. Additional insight was provided based on knowledge of modeling and simulation activities beyond those reported as part of the study publication.
- Updated by Joy Ku on April 24, 2020. Added categories for defining context (Rule 1).
- Edited and formatted by Ahmet Erdemir on July 25, 2020 as supplementary material for related publication [1].

## Disclaimer

THIS EXAMPLE DOES NOT NECESSARILY DEMONSTRATE THE LEVEL OF DETAIL REQUIRED TO FULLY SATISFY THE COMMITTEE’S CREDIBILITY GUIDELINES [1]. THE GRANULARITY OF HOW EACH RULE IS APPLIED IS SOLELY DEPENDENT ON THE NEEDS OF EACH MODELING AND SIMULATION PROJECT.

**Rule 1 – Define context clearly**

- Domain of Use: Fundamental research.
- Use Capacity: The study was interested in assessing variants of hyperexcitability in an exploratory manner. Therefore there were no pre-defined and clear clinical context but an openness to exploration of cortical activation for dystonia and seizures.
- Strength of Influence: Unclear.

**Rule 2 – Use contextually appropriate data**

- Data used to develop the model were taken from a large number of sources including different species, different preparations (slice, cell culture, in vivo, ex vivo), different age animals, different states, different conditions.
- None of the data were taken from the clinical disorders in question (limitations of human experimentation).

**Rule 3 – Evaluate within context**

- The model lacked the major output for dystonia validation, motor output, so it could not be evaluated in proper context.
- Epilepsy context was somewhat more clear since based on cortical activity level.
- Beta activation in cortex was used as a dystonia evaluation surrogate.

**Rule 4 – List limitations explicitly**

- Model does not contain representations of spinal cord or limb.
- Model is lacking many relevant pharmacological parameters, particularly with respect to role of neuromodulators (known unknown), brain states (less known unknown) and metabolic parameters.

**Rule 5 – Use version control**

- Mercurial (hg) version control management tool was used for all simulations as well as for manuscript and figure versioning.

**Rule 6 – Document appropriately**

- Model is available with README on ModelDB (189154) [4].
- Parameter provenance and documentation are available in manuscript. Strong provenance in regard to assessment in context (brain modeling/computational neuroscience).

**Rule 7 – Disseminate broadly**

- Model code was made available on ModelDB [4] for manuscript reviewers on request. Model is freely available after publication.
- Model and data were disseminated via meetings.
- Manuscript was published in an open-source journal.
- Previous models have been used and modified by others.

**Rule 8 – Get independent reviews**

- Model was reviewed for run-capability on multiple platforms by ModelDB [4] curator (Tom Morse) prior to release.
- Model was reviewed by manuscript reviewers but was not run and examined in detail.

**Rule 9 – Test competing implementations**

- No other implementation was attempted. Partially comparable implementations for epilepsy in neocortex do exist (e.g., Traub models, authors’ prior models) and activity patterns could be compared.

**Rule 10 – Conform to standards**

- Conformed to standards for ModelDB [4] submission and to best practice standards as taught in NEURON [5] courses.
- Simulation workflows, data collection, data processing, data reporting conformed to practices generally accepted by the Computational Neuroscience community.

## References

1. Erdemir A, Mulugeta L, Ku JP, Drach A, Myers JG, Horner M, Morrison T, Vadigepalli R, Lytton WW. Credible practice of modeling and simulation in healthcare: ten rules from a multidisciplinary perspective. J Transl Med.
2. Neymotin SA, Dura-Bernal S, Lakatos P, Sanger TD, Lytton WW. Multitarget multiscale simulation for pharmacological treatment of dystonia in motor cortex. Front Pharmacol. 2016 Jun 14;7:157. doi: 10.3389/fphar.2016.00157.
3. Neymotin2016 - cpms [[Internet]. [cited 2020 Jul 25]. Available from:](http://paperpile.com/b/KXjwuZ/O1dK) <https://simtk.org/plugins/moinmoin/cpms/Neymotin2016>
4. ModelDB: Home [[Internet]. [cited 2020 Jul 25]. Available from:](http://paperpile.com/b/KXjwuZ/O1dK) <https://senselab.med.yale.edu/ModelDB/>
5. NEURON | empirically-based simulations of neurons and network of neurons [[Internet]. [cited 2020 Jul 25]. Available from:](http://paperpile.com/b/KXjwuZ/O1dK) <https://neuron.yale.edu/neuron/>

# Supplementary Material - Example 4

This document is prepared as supplementary material for the publication titled “Credible practice of modeling and simulation in healthcare: ten rules from a multidisciplinary perspective” [1].

## Study

Modeling the dynamics of human liver failure post liver resection [2].

## Modeling Area

Ordinary differential equation-based modeling of liver regeneration.

## Background

Liver resection is an important clinical intervention to treat liver disease. Following liver resection, patients exhibit a wide range of outcomes including normal recovery, suppressed recovery, or liver failure. The objective was to study these distinct patient outcomes post resection and determine the processes that are accountable for liver failure.

This study utilized a previously developed computational model of liver regeneration [3] and fine-tuned the parameters based on liver volumetric data from patients that underwent liver resection. The simulations focused on the model parameters that control the cell death process and utilized a virtual patient cohort that was created by sampling across the parameter space involving metabolic load and cell death sensitivity.

## Analysis History

A living version of this example can be found online [4].

- Original analysis report by Rajanikanth Vadigepalli (study co-author), September 3, 2018. Additional insight was provided based on knowledge of modeling and simulation activities beyond those reported as part of the study publication.
- Updated by Joy Ku on April 23, 2020. Added categories for defining context (Rule 1).
- Edited and formatted by Ahmet Erdemir on July 25, 2020 as supplementary material for related publication [1].

## Disclaimer

THIS EXAMPLE DOES NOT NECESSARILY DEMONSTRATE THE LEVEL OF DETAIL REQUIRED TO FULLY SATISFY THE COMMITTEE’S CREDIBILITY GUIDELINES [1]. THE GRANULARITY OF HOW EACH RULE IS APPLIED IS SOLELY DEPENDENT ON THE NEEDS OF EACH MODELING AND SIMULATION PROJECT.

**Rule 1 – Define context clearly**

- Domain of Use: Clinical research.
- Use Capacity: Model is intended to provide additional data about 1) different patient outcomes and 2) the processes that may account for liver failure post liver resection.
- Strength of Influence: The model and simulations are intended to identify processes that may be accountable for liver failure and generate hypotheses for further investigations. It is not being developed to predict which individuals will experience liver failure.

**Rule 2 – Use contextually appropriate data**

- Model was built on a previous model that was based on extensive rodent data.
- Human data used to tune the present model was taken from a published clinical data set.

**Rule 3 – Evaluate within context**

- Model simulations were evaluated using global sensitivity analysis and for consistency with available clinical data to match the range of observed timescales of liver failure.
- Parameters chosen for variation in the model were based on sensitivity analysis results and biological relevance.

**Rule 4 – List limitations explicitly**

- Model does not contain a representation of liver metabolic functions that are altered during liver failure after a resection (known unknown), and whole-body physiological parameters that control liver response to resection (less known unknown). These unaccounted mechanisms affect how metabolic load varies across patients, and likely alter the predictions on key factors controlling the timescale of liver failure.

**Rule 5 – Use version control**

- Manual versioning was used and it was difficult to track changes.

**Rule 6 – Document appropriately**

- Model equations and code are available in the publication supplement.
- Partial parameter provenance and documentation corresponding to the parameters that were tuned are available in manuscript.

**Rule 7 – Disseminate broadly**

- Model code was included in the manuscript supplement during review.
- Model is freely available after publication in an open access journal.
- Previous models have been used and modified by others.

**Rule 8 – Get independent reviews**

- Model was reviewed by manuscript reviewers.
- Model was implemented on multiple platforms by an independent laboratory member who was not involved with the project, prior to manuscript submission.

**Rule 9 – Test competing implementations**

- No other was implementation attempted.
- More detailed models exist that account for multiple cell types, and have been tuned to rodent data. However, human data to tune such detailed models is lacking.

**Rule 10 – Conform to standards**

- Model is Systems Biology Markup Language [5] and in Matlab code.
- Simulation workflows, data collection, processing, and reporting conformed to practices generally accepted by the computational biology community.

## References

1. Erdemir A, Mulugeta L, Ku JP, Drach A, Myers JG, Horner M, Morrison T, Vadigepalli R, Lytton WW. Credible practice of modeling and simulation in healthcare: ten rules from a multidisciplinary perspective. J Transl Med.
2. Verma BK, Subramaniam P, Vadigepalli R. Modeling the dynamics of human liver failure post liver resection. Processes (Basel). 2018 Aug;6(8):115. doi: 10.3390/pr6080115.
3. Cook D, Ogunnaike BA, Vadigepalli R. Systems analysis of non-parenchymal cell modulation of liver repair across multiple regeneration modes. BMC Syst Biol. 2015 Oct 22;9:71. doi: 10.1186/s12918-015-0220-9.
4. Verma2018 - cpms [[Internet]. [cited 2020 Jul 25]. Available from:](http://paperpile.com/b/KXjwuZ/O1dK) <https://simtk.org/plugins/moinmoin/cpms/Verma2018>
5. Main Page - SBML.caltech.edu [[Internet]. [cited 2020 Jul 25]. Available from:](http://paperpile.com/b/KXjwuZ/O1dK) <http://sbml.org/Main_Page>
