# Supplementary material for: Credible practice of modeling and simulation in healthcare: ten rules from a multidisciplinary perspective
Source: J Transl Med. 2020 Sep 29;18:369. doi: 10.1186/s12967-020-02540-4 (PMC7526418; doi:10.1186/s12967-020-02540-4)
Supplement: Supplementary file 2 — Additional file 2: Supplementary Tables: A collection of tables intended to provide the interested reader additional insight into the ten rules’ development details and the benefit of applying the ten rules throughout a model and simulation life cycle. [file 12967_2020_2540_MOESM2_ESM.docx]

**Supplementary Materials**

**Table SM-1:** The three task teams had 3 members from the Executive Committee and 4 members from the Advisory Council. Note that no member was on more than one team.

| **Task Teams** | **Mathematics and**  **Computation** | **End Users** | **Standards and**  **Guidelines** |
| --- | --- | --- | --- |
| **Background of team members** | scientists and engineers from academia, industry, government agencies, and independent | clinicians, scientists, and engineers from academia, industry, government agency, and healthcare organizations | scientists, engineers, directors, and managers from industry and government agencies |
| **Representation for modeling and simulation stakeholders** | fundamental and applied researchers, including commercial developers of modeling and simulation related products for healthcare | focused on clinical research and practice | focused on establishing professional and/or industry practice guidelines, regulations, and standards |

**Table SM-2:** The 32 proposed rules that were surveyed by the global stakeholder community. The three repeated rules are listed at the end of the list with a designation “R-#”. The “#” after the “R” indicates the number of the original rule that is being duplicated. These duplicate questions were included in the survey to establish the consistency of the responses from each survey respondent.

| 1 | Engage potential end-user base. | 19 | Perform uncertainty estimation/quantification within context of use. |
| --- | --- | --- | --- |
| 2 | Make the modeling and simulation results reproducible. | 20 | Perform numerical error estimation/quantification within context of use. |
| 3 | Develop the modeling and simulation with the end-user in mind. | 21 | Get the modeling and simulation reviewed by independent users, developers, and members of the intended stakeholder community. |
| 4 | Use appropriate data, e.g., for input, validation, verification. | 22 | Explicitly list limitations of the modeling and simulation. |
| 5 | Explicitly identify experimental scenarios that illustrate when, why, and how the modeling and simulation is false or not applicable. | 23 | Make it easy for anyone to repeat and/or falsify your results. |
| 6 | Use competition of multiple modeling and simulation implementation methods to check and balance each other. | 24 | Use consistent terminology or define your terminology. |
| 7 | Document the development and use of modeling and simulation appropriately. | 25 | Verify the modeling and simulation processes within context of use. |
| 8 | Use version control, i.e., to track different revisions of the model. | 26 | Report appropriately, i.e., to allow reproducibility, to assess reliability, and to establish accountability. |
| 9 | Be a discipline specific example of good practice. | 27 | Define the context in which the modeling and simulation is intended to be used. |
| 10 | Use data that can be traced back to the origin of source. | 28 | Conform to discipline-specific standards. |
| 11 | Disseminate whenever and whatever is possible, e.g., source code, test suite, data. | 29 | Disclose conflict of interests. |
| 12 | Validate the modeling and simulation activity within the context of use. | 30 | Adopt and promote standard operating procedures. |
| 13 | Perform sensitivity analysis within the context of use. | 31 | Document your code. |
| 14 | Define the modeling and simulation evaluation metrics in advance. | 32 | Use simulation software with established reliability |
| 15 | Make your code readable. | R-28 | Follow discipline-specific guidelines and standards whenever possible. |
| 16 | Provide user instructions whenever possible and applicable. | R-32 | Use credible, e.g., verified, solvers (code, software, applications). |
| 17 | Provide examples of use. | R-22 | Provide clear descriptions of limitations. |
| 18 | Learn from specialized and broadly applicable guidelines for good practice. |  |  |

Table SM-3: Mapping the Ten Rules for Credible Practice in Models and Simulations in Healthcare to the generalized model and simulation life-cycle. Each check indicates that activities in that life-cycle component contributes to pursuing credible practice and can be communicated to user community and other stakeholders.

| **Credible Practice Rule** | **Communicate Life-Cycle Components** | | | | | |
| --- | --- | --- | --- | --- | --- | --- |
|  | **Model Development** | **Model Calibration** | **Model Benchmarking** | **Accreditation** | **Intended Use** | **Historical Use / Reuse** |
| **Define context clearly** | **✔** | **✔** | **✔** | **✔** | **✔** | **✔** |
| **Use contextually appropriate data** | **✔** | **✔** | **✔** |  | **✔** | **✔** |
| **Evaluate within context** |  | **✔** | **✔** | **✔** | **✔** |  |
| **List limitations explicitly** | **✔** | **✔** | **✔** | **✔** | **✔** | **✔** |
| **Use version control** | **✔** | **✔** | **✔** | **✔** | **✔** | **✔** |
| **Document appropriately** | **✔** | **✔** | **✔** | **✔** | **✔** | **✔** |
| **Disseminate broadly** | **✔** | **✔** | **✔** | **✔** | **✔** | **✔** |
| **Get independent reviews** | **✔** | **✔** | **✔** | **✔** |  |  |
| **Test competing implementations** |  | **✔** | **✔** | **✔** | **✔** |  |
| **Conform to standards** | **✔** | **✔** | **✔** | **✔** | **✔** | **✔** |

Table SM-4: Examples of the benefits of and pitfalls related to acknowledgment (or lack thereof) of the Ten Rules for credible practices in modeling and simulation in healthcare. These should not be considered an exhaustive list, as individual model implementations may have specific benefits and pitfalls.

| **Credible Practice Rule** | **Benefit to Including** | **Pitfall to Excluding** |
| --- | --- | --- |
|  |  |  |
| **Define context clearly** | Defines purpose and expectations of model performance and utility to user community | Lack of clarity of relevance and intensity of testing and other credibility factors to build confidence in the user community |
| **Use contextually appropriate data** | Traceability of all sources of data and relevance to model utility for the user community | Community cannot discount a lack of quality informing the model or the use of inappropriate data |
| **Evaluate within context** | Communicates the strength of testing and internal scrutiny performed and its relevance to the user community | Inability of the user community to determine if the testing is appropriate, performed with a suitable referent, or extensive enough to address intended and future use. |
| **List limitations explicitly** | Informs the user community to the extents the model can be applied per situations | Exposes the community to potential mis-use of the model, or use out of context or outside the range in which it is intended |
| **Use version control** | Associates model and simulation products and historical use to the specific version of the model; establishes provenance to data | Hinders the user community from accurate interpretation, repeatability, and debugging of the historical simulation predictions. |
| **Document appropriately** | Establishes products and evidence which directly communicate all aspects of the model with sufficient fidelity to allow review and assessment of model and model development process and reproduce modeling and simulation steps | Insufficiencies in this area prevent the community from establishing the suitability of this model in its context of use or future use, regardless of its status in other credibility factors. It further impedes the ability to reproduce the model or replicate the model results. |
| **Disseminate broadly** | Allows the user community to access, inspect, test, and comment on the model and application, improving the developers ability to address model issues and eventual intended use. Augments the potential for reuse. | Inhibits community buy-in on the model and its products, as well as limits the developers ability to receive constructive feedback from the community, thus potentially limiting. Severely diminishes reuse. |
| **Get independent reviews** | Provides a level of confidence that an unbiased assessor has critically reviewed all aspects of the credibility evidence and provided the findings for developer disposition and community review. | Decision makers that lack in depth knowledge to the model and simulation cannot assess the suitability of the model for their use or determine the weight to attribute to the model predictions |
| **Test competing implementations** | Represents a comparative metric to operational models with which the user community has familiarity and has similar context of use. Serves to illustrate the application of both models. | User community lacks insight into the quantitative benefits of this model with respect to other similar models which may impede appropriate use and adoption. |
| **Conform to standards** | Ensures the user community that a minimum level of rigor has been followed with respect to that expected in the discipline(s) associated with the model development and intended use. Enhances comprehensibility and interoperability of modeling and simulation by-products. | The user community cannot easily assess if the level rigor in the model development and representation of modeling and simulation products meets the discipline standards without substantial audits by discipline experts and/or ad hoc treatment of outputs. This impacts the confidence in the model to adequately address the intended use. Model exchange and reuse can be diminished. |
